# Microangiopathy in temporal lobe epilepsy with diffusion MRI alterations and cognitive decline

**DOI:** 10.1007/s00401-024-02809-8

**Published:** 2024-10-08

**Authors:** Joan Liu, Lawrence Binding, Isha Puntambekar, Smriti Patodia, Yau Mun Lim, Alicja Mryzyglod, Fenglai Xiao, Shengning Pan, Remika Mito, Jane de Tisi, John S. Duncan, Sallie Baxendale, Matthias Koepp, Maria Thom

**Affiliations:** 1 Department of Clinical and Experimental Epilepsy, Department of Neuropathology, UCL Queen Square Institute of Neurology, London, WC1N 3BG UK; 2 Department of Neuroscience, University of Westminster, London, UK; 3 Centre for Medical Image Computing, Department of Computer Science, UCL, London, UK; 4 Department of Statistical Science, University College London, Gower St., London, UK; 5 Department of Neuroscience and Mental Health, Florey Institute of Neuroscience and Mental Health, University of Melbourne, Victoria, Australia

**Keywords:** White matter, Temporal lobe epilepsy, Microangiopathy, Diffusion MRI, Fixel-based analysis, Cognitive decline

## Abstract

White matter microvascular alterations in temporal lobe epilepsy (TLE) may be relevant to acquired neurodegenerative processes and cognitive impairments associated with this condition. We quantified microvascular changes, myelin, axonal, glial and extracellular-matrix labelling in the gyral core and deep temporal lobe white matter regions in surgical resections from 44 TLE patients with or without hippocampal sclerosis. We compared this pathology data with in vivo pre-operative MRI diffusion measurements in co-registered regions and neuropsychological measures of cognitive impairment and decline. In resections, increased arteriolosclerosis was observed in TLE compared to non-epilepsy controls (greater sclerotic index, *p* < 0.001), independent of age. Microvascular changes included increased vascular densities in some regions but uniformly reduced mean vascular size (quantified with collagen-4, *p* < 0.05–0.0001), and increased pericyte coverage of small vessels and capillaries particularly in deep white matter (quantified with platelet-derived growth factor receptorβ and smooth muscle actin, *p* < 0.01) which was more marked the longer the duration of epilepsy (*p* < 0.05). We noted increased glial numbers (Olig2, Iba1) but reduced myelin (MAG, PLP) in TLE compared to controls, particularly prominent in deep white matter. Gene expression analysis showed a greater reduction of myelination genes in HS than non-HS cases and with age and correlation with diffusion MRI alterations. Glial densities and vascular size were increased with increased MRI diffusivity and vascular density with white matter abnormality quantified using fixel-based analysis. Increased perivascular space was associated with reduced fractional anisotropy as well as age-accelerated cognitive decline prior to surgery (*p* < 0.05). In summary, likely acquired microangiopathic changes in TLE, including vascular sclerosis, increased pericyte coverage and reduced small vessel size, may indicate a functional alteration in contractility of small vessels and haemodynamics that could impact on tissue perfusion. These morphological features correlate with white matter diffusion MRI alterations and might explain cognitive decline in TLE.

**Supplementary Information:**

The online version contains supplementary material available at 10.1007/s00401-024-02809-8.

## Introduction

There is a growing body of evidence that microvascular dysfunction occurs in epilepsy of potential detriment to both seizure control and its comorbidities [51, 55]. Pathological alterations of the white matter in epilepsy, including temporal lobe epilepsy (TLE), have demonstrated small vessel disease (SVD), abnormal vascular collagen deposition, angiogenesis and altered pericytes and reduction of white matter axons and myelination [2, 9, 12, 20, 21, 27]. There is evidence that microvascular pathology in epilepsy impacts on white matter haemodynamics, blood brain barrier (BBB) function and myelin integrity [68]. Experimentally, ictal contractions of pericytes have been shown to induce vasospasm and ischaemia-related neurodegeneration [37] and pericyte injury in epilepsy leads to neurovascular decoupling [51] with a dynamic reorganisation proportional to seizure severity [3]. BBB dysfunction in experimental seizures [42], has also been demonstrated following a single seizure in patients [61]. Furthermore, SVD is itself associated with late onset seizures [15], with proposed mechanisms for epileptogenesis including impaired cerebral perfusion and disruption of subcortical networks [78]. Our aim in a large surgical TLE series was to address alterations of the white matter microvasculature in relation to myelination.

Memory impairment and cognitive dysfunction are a significant co-morbidity in TLE [6]. Pathology studies have mainly focused on acquired hippocampal degeneration [38, 70, 82] but this does not fully explain the clinical picture; a contribution of vascular dysfunction to memory performance was recently shown but remains relatively under-investigated [56]. Degenerative small vessel disease of the white matter represents a leading causes of dementia including subcortical vascular dementia [46] and contributes to Alzheimer’s disease [33]. Furthermore, diffusion weighted MRI (DWI) of white matter in epilepsy shows widespread reduction of fractional anisotropy (FA) and increased mean diffusivity (MD) including in TLE (Hatton 2020); studies have identified changes specifically in the subcortical white matter [39, 72] and shown a link with cognitive phenotypes, memory and language impairment [57]. Fixel-based diffusion analysis (FBA) has an additional potential to decipher microstructure alterations in the complex fibre organization of superficial white matter [13]. We hypothesized that SVD in TLE is of relevance to memory impairment and impacts on the DWI alterations in the white matter. In this cohort of operated TLE patients with well-characterized pre-operative neurocognitive function, our aim was to explore any regional pathological differences in white matter microangiopathy in superficial and deep white matter, and to compare this within-vivo pre-operative MRI diffusion measures (DWI and FBA) in similar regions.

## Materials and methods

### Case selection

Cases were obtained from the Epilepsy Society Brain and Tissue Bank (ESBTB) at University College London Queen Square Institute of Neurology which has ethical approval and all patients consented for use of tissue and linked clinical data and MRI in research. We included 44 adult patients who had undergone an anterior temporal lobe resection with hippocampectomy for the treatment of drug-refractory TLE with a pathological and MRI diagnosis of either hippocampal sclerosis (HS, *n* = 33, left *n* = 21) or non-lesional without HS (non-HS, *n* = 11, left *n* = 6). For 24 of those 44 patients, DWI data was available. Tumours, cortical dysplasia, cavernomas, other focal lesions and patients with prior surgery were excluded; some cases showed patchy cortical neuronal loss and gliosis but without a specific diagnosis. 15/44 cases had stereo-EEG investigations prior to resection but electrode tracks were excluded from the sections evaluated. Control cases (post-mortem samples) were selected from consented cases donated to the ESBTB and the MRC Edinburgh Brain and Tissue Bank (EBTB) who were non-epilepsy controls (NEC; *n* = 28) with no neurological disease or neuropathology following brain examination and epilepsy controls (EPC; *n* = 8) with no lesional brain pathology at post-mortem, no history of TLE, who had died from sudden and unexpected death in epilepsy (SUDEP). Controls were sex matched but the NEC were older than the TLE group, summarised in Table 1 with details in supplemental Table 1 including age ranges, any vascular disease risk factors and cause of death. For gene expression studies, a cohort of 36 cases was selected including TLE with HS (*n* = 16) and non-lesional TLE (*n* = 6), non-lesional frontal-lobe epilepsy (*n* = 6), non-lesional post-mortem EPC (from ESBTB, *n* = 4) and NEC (from EBTB, *n* = 4). The 22 TLE cases were selected to represent a range of ages at surgery (10 < 40 years at surgery; 12 > 40 years at surgery); we excluded cases with prior stereoencephalography (S-EEG) investigations to avoid inclusion of electrode track scars in white matter tissue (Table 1). Ten of the TLE cases were also in the DWI cohort (see Supplemental Fig. 1 for flowchart of study group selection).

**Table 1:** Summary data of the cases selected for studies

Study	Group (N = number of cases)	Tissue types	Left: Right sides studied	Mean age at surgery or death (range) years	Mean age at onset of epilepsy (range) years	Duration of epilepsy years	Seizure types FAS/FIAS/GS/SE (% cases)	Sex M: F	S-EEG prior to resection; number	ASM Mean number trialled (range)	Seizure-free outcome (ILAE) 2 years/5 years (mean; range)	Period of tissue sample collection
Pathology studies	TLE-HS (N = 33)	S-FFPE	21: 12	39.4 (21–60)	12.0 (0.7–37)	27.3 (4–47)	42/93/90/18	16: 18	9	7.6 (2–16)	2(1–5)/ 2 (1–4)	2003–2021
	TLE-No HS (N = 11)	S-FFPE	5: 6	37.6 (24–55)	17.3 (7–51)	20.7 (1–40)	54/81/81/18	6: 5	6	6.8 (1–11)	2.3 (1–5)/ 2.6 (1–5)	2011–2019
	NEC (N = 28)	PM-FFPE	25: 25	47.5 (23–90)	N/A	N/A	N/A	15: 13	N/A	N/A	N/A	1998–2019
	EPC (N = 8)	PM-FFPE	8: 8	29.87 (16–41)	15* (5–26)	14* (1–28)	na	5: 3	N/A	na	N/A	2013–2019
Gene expression studies	TLE-HS (N = 16)	S-FS	8:8	43.8 (20–60)	14.9 (3–50)	28.8 (7–47)	–	6: 10	0	–	–	2011–2021
	TLE-No HS (N = 6)	S-FS	1: 5	37.5 (21–50)	11.8 (12–25)	25.6 (6–40)	–	4: 2	0	–	–	2017–2019
	FLE (non- lesional) (N = 6)	S-FS	2; 4	35.5 (23–47)	17.1 (5–41)	18.3 (6–36)	–	4: 2	0	–	–	2012–2015
	NEC (N = 4)	PM-FS	na	43 (33–58)	N/A	N/A	N/A	4: 0	N/A	N/A	N/A	2014–2016
	EPC (N = 4)	PM-FS	na	44 (33–57)	17.6* (1–31)	28.3* (2–47)	na	2: 2	N/A	–	N/A	2015–2021
DWI	TLE CASES	N/A	15: 10	38.7 (21–60)	12.4 (0.7–37)	26.2 (7.2–47)	32/92/88/28	10: 15	–	8 (3–16)	2.1 (1–5)/ 2.2(1–5)	N/A
	CONTROLS	N/A		40 (19–66	N/A	N/A	N/A	26: 44	N/A	N/A	N/A	N/A

*ASM* antiseizure medications (further detailed in supplemental Table 1), *EPC* epilepsy controls, *FAS* focal aware seizures, *FFPE* formalin-fixed paraffin-embedded, *FIAS* focal impaired awareness seizures, *FLE* frontal lobe epilepsy, *FS* Frozen (− 70 °C) sample, *GS* generalised (tonic–clonic) seizures documented, now termed focal to bilateral tonic–clonic seizures, *NEC* non-epilepsy controls, *PM* post mortem, *S-EEG* stereo electroencephalogram, *S* surgical, *SE* history of prior status epilepticus, *TLE* temporal lobe epilepsy. *N/A* not applicable, *na* not available and *–* data not evaluated

*For post-mortem cases precise clinical information on age of seizure onset and types is missing for some cases (see supplemental Table 1). For NEC cases, all autopsies were performed within 24 h after death and fixed in neutral buffered formalin for no more than one week. No remarkable abnormalities were observed in white matter of any cases during standard neuropathology assessment using routine histological stains and immunohistochemical panels consisting of neuronal, astrocytic and microglial markers except for one PM case which had small area of white matter scarring. Clinical records on patients’ seizure history, including age of onset, seizure types (generalised convulsive, focal) and risk factors for arteriolosclerosis can be found in supplemental Table 1 and diagram of cohort section in Supplemental Fig. 1.

### Tissue preparation for pathology measures

A tissue block representing a coronal section through the superior (STG), middle (MTG), inferior (ITG) and fusiform temporal gyri (FG) was selected from surgical cases at 1.5 cm from the temporal pole. The temporal gyri had been marked following surgery for gyral orientation and correlation with the pre- and post-operative MRI was also used for further anatomical alignment in some cases. The tissue was formalin-fixed, routinely processed, and serial sections cut at 5-micron intervals. For control cases, formalin-fixed paraffin-embedded tissue blocks from both hemispheres, from STG and MTG were used when available, and similarly sectioned. This resulted in a final group of 102 temporal lobe samples for investigation, although not all gyral regions were available in all cases and controls (see supplemental Table 1 for regions available in each case).

#### Vascular sclerosis measurements

We used the sclerotic index (SI), a standard method used in vascular dementia to quantify degenerative small vessel pathology on H&E sections. With NDP.view3 software (Hamamatsu Photonics) the internal, external and perivascular space (PVS) diameters in two perpendicular axis were measures in the 20 most sclerosed arterioles across the white matter and a mean SI for each case recorded. (Supplemental Fig. 1A and methods).

### Immunostaining analysis and quantitation

A panel of antibodies for white matter structures and vessels was used and standard immunohistochemistry was carried out. The rationale for selection is summarized in Table 2 and staining protocols further detailed in the supplemental methods. Double-labelling immunofluorescence for PDGFRβ and smooth muscle actin (SMA) was carried out on 4 TLE cases and 4 controls (Supplemental Table 1 and methods). Slides were scanned with a NanoZoomer S360 Digital slide scanner (C13220-01, Hamamatsu Photonics K.K) at 40 × magnification as a single z-stack and immunofluorescence sections with a Hamamatsu NanoZoomer S60 Digital slide scanner (C13210-04, Hamamatsu Photonics K.K). Using QuPath software (Version 0.4.1, [4]) white matter regions of Interests (ROIs) were defined from each available gyrus, further divided into ‘core’ and ‘deep’ white matter using an anatomical boundary of straight line joining the bottom of two adjacent gyri (Supplemental Fig. 2). The core white matter therefore represented the superficial and subcortical ‘U’ fibres but excluding cortical grey matter; any tissue artefacts were also excluded. Using QuPath intensity thresholds were set at three levels for each marker (low, medium and high thresholds) and optimal thresholds selected to maximize specific and minimizing non-specific detection (see supplemental Fig. 3), keeping thresholds constant across all cases and controls. A labelling index was calculated for each ROI (i.e. a field fraction of total labelled area). In addition, cell density measurements on NeuN and Olig2 sections were determined using Qupath Cellpose extension (https://github.com/BIOP/qupath-extension-cellpose) [50, 67] (Supplemental Fig. 3, Supplemental methods).

**Table 2:** Immunohistochemistry (IHC) panel, method and quantitative analysis conducted

Group	IHC marker	Expression patterns; rationale for use in study	Antibody clone; source; dilution; IHC method	Quantitative analysis
MYELIN AND AXONS	Myelin associated glycoprotein (MAG)	Type 1 transmembrane glycoprotein on the inner membrane of myelin sheaths; susceptible to reduced tissue oxygenation as marker of tissue hypoperfusion/small vessel disease [44]	Rabbit monoclonal ab277524; Abcam, UK; 1:2500; ER2-20 min; Leica Bondmax	Automated whole slide image analysis (Qupath) [4]—Labelling Index in ROI
	Phospho-lipid protein-1 (PLP)	Myelin transmembrane domain protein/binds myelin sheath and interacts with MAG; in comparison to MAG relatively resistant to tissue hypoperfusion [44]	Rabbit polyclonal HPA004128; Cambridge Biosciences; 1: 500; ER1-20 min; Leica Bondmax	Automated whole slide image analysis (Qupath)—Labelling Index in ROI
	Neurofilament Light chain (NF-L)	Type IV intermediate filament (MW 68 KDa); expression, phosphorylation reflects axon calibre, myelination and conduction velocity [23]; loss may reflect axonal injury/depletion [84]	Mouse monoclonal Phosphorylated Neurofilament cocktail, DAKO/Cappel (NFC); M0762; 1:500; ER2-20 min; Leica Bondmax	Automated whole slide image analysis (Qupath) – Labelling Index in ROI
	Neurofilament Medium chain (NF-M)	Type IV intermediate filament (MW 150 KDa); as for NF-L	Mouse monoclonal Neurofilament phosphorylation (NFP)—also reacts with HF-H; MAB1592; Merck; 1:3200; ER1-20 min; Leica Bondmax	Automated whole slide image analysis (Qupath)—Labelling Index in ROI
	Neurofilament Heavy chain (NF-H)	Type IV intermediate filament (MW 190–210 KDa); as for NF-L	Mouse monoclonal SMI31 Phosphorylated neurofilament 801,601; Sternberger/Biolgend; 1:5000; no antigen retrieval; Leica Bondmax	Automated whole slide image analysis (Qupath) – Labelling Index in ROI
GLIA AND MATRIX	Neuronal nuclear antigen (NeuN)	Mature Neuronal marker	Rabbit polyclonal ab104225; Abcam; 1:500; ER1-20 min; Leica Bondmax	Qupath/cellpose for automated cell density
	Oligodendrocyte transcription factor 2 (Olig2)	Oligodendroglial lineage marker	Mouse monoclonal MABN50 211F1.1; Sigma Aldrich; 1:400; ER2-20 min; Leica Bondmax	Qupath/cellpose for automated cell density
	Ionized calcium-binding adaptor molecule 1 (Iba1)	Resting and activated microglia	Rabbit polyclonal 019–19741; Fujifilm WAKO; 1:1000; ER2- 30 min; Leica Bondmax	Automated whole slide image analysis (Qupath) – Labelling Index in ROI
	Tenascin C	Extracellullar matrix protein; roles in tissue repair, upregulated in gliosis and angiogenesis and experimental epilepsy [29]	Rabbit monoclonal ab108930; Abcam; 1:300; citrate buffer—12 min; manual IHC	Automated whole slide image analysis (Qupath)—Labelling Index in ROI
ANGIOGENESIS AND SMALL VESSELS	Platelet derived growth factor receptor-Beta (PDGFRβ)	Marker for vascular pericytes [65]	Rabbit monoclonal Ab32570; Abcam; 1:500; ER2 – 20 min; Leica Bondmax	Automated whole slide image analysis (Qupath) – Labelling Index in ROI Manual measurements of type 1 and 2 vessels (see text for details)
	Collagen 4 alpha 1 (COL4)	Vascular basement membrane, major structural component	Rabbit polyclonal Ab189408; Abcam; 1:100; ENZ1—15 min; Leica Bondmax	Qupath/automated total vascular density and size measurements
	Smooth muscle actin (SMA)	Vascular smooth muscle cell and pericyte marker [65]	Mouse monoclonal, 1A4, Agilent DAKO; 1:500; no antigen retrieval; Ventana Discovery Ultra	Qupath/semi-automated vascular density and size measurements of type 1 and 2 vessels

Further detail on methods is available in supplementary materials files

#### PDGFRβ analysis, vascular structures and glia

PDGFRβ highlighted pericytes and perivascular cells in immediate proximity to small vessels and capillaries with lumen, in addition to multipolar parenchymal cells not associated with vessels. PDGFRβ was therefore manually quantified for vascular structures in addition to automated labelling index for total PDGFRβ labelling (See Supplemental methods). PDGFRβ + vascular structures of all size were classified into Type 1 or Type 2 vessels, according to either complete or partial vascular PDGFRβ + pericyte coverage, respectively (Supplemental Fig. 1B) and also categorized as small capillaries (< 5 microns), capillaries (5 to ≤ 10 microns), small arterioles (10 to ≤ 25 microns) and arterioles (> 25 microns) and the density and proportion of vessel types in each ROI calculated based on their tangential diameters.

#### Col4 and SMA vascular structures

To evaluate the density of white matter COL4 + vessels and diameters, an automated method was developed in Qupath (YML) and vessel density/mm2 and median vascular diameter for each ROI calculated (Supplemental Fig. 1C–G). A similar but modified approach was carried out for SMA but, as perivascular cell labelling was often discontinuous, SMA vessels were also categorized as Type 1 if the labelling was circumferential or Type 2 if discontinuous (Supplemental Fig. 4 and methods). For PDGFRβ/SMA double labelling, 2mm2 ROI were quantified using NDP.view2 software in STG and MTG core and deep white matter for the density of double and single labelled vessels and vessel diameters, distinguishing type 1 or 2 SMA + vessels.

### White matter gene expression data

Total RNA was isolated from the deep white matter of 36 cases (the gyral core regions were not separately analysed) and processed for Nanostring nCounter analysis using NanoString Neuropathology Gene Expression panel (XT Hs NeuroPath CSO; NanoString Technologies, Washington, USA), which target genes involved in neuroplasticity, development and aging, neuroinflammation, metabolism and maintenance of structural integrity pathways (Supplemental methods). Visualisation of data through constructions of heatmaps, correlative matrices and graphs were achieved using nSolver software (version 4.0, NanoString, Washington, USA). Multivariate linear modelling was performed to identify significant differentially expressed genes in different comparative groups divided as follows: Pathology (HS/Non-lesional), age of Surgery (under/over 40 years), TLE (surgical/PM controls).

### Diffusion MRI analysis

The study included 24 patients with pre-operative DWI, (including 10 with gene expression data, Supplemental Table 1), and was compared to 70 age and sex matched healthy controls from a subset of a previous study [77]. Diffusion data were collected and methods are provided in detail in supplemental files. In brief, the acquisition parameters were: 3 T GE Discovery MR750 was used to collect a 3D T1-weighted sequence (MPRAGE) and multi-shell dMRI (2 mm isotropic resolution, gradient directions: 11, 8, 32, and 64 at b-values: 0, 300, 700, and 2500 s/mm2; ∂/Δ = 21.5/35.9 ms, TE/TR = 74.1/7600 ms). MRI images were aligned with pathology resection specimens and using Freesurfer (version 7, [19]), the white matter was parcellated from the T1-weighted image into superficial and deep STG, MTG and ITG regions. The DWI data were modelled using DTI to extract tensor-based metrics, including the mean diffusivity (MD), fractional anisotropy (FA), axial diffusivity (AD), and radial diffusivity (RD).

#### Fixel-based analysis (FBA)

DWI data were also analysed using the FBA framework [53]. In brief, a measure of fibre density and cross-section (FDC) was obtained at each white matter fixel in the study-specific population template space (whereby ‘fixel’ refers to a specific fibre population within a single image voxel). This FDC metric combines microstructural and morphological information, such that it is sensitive to changes in the density of fibres passing in a particular direction within a given voxel, as well as the changes in the cross-section of fibre bundles that traverse multiple image voxels. Connectivity-based smoothing was performed on fixel-based measures for all participants [52].

### Clinical data and neuropsychometry

Clinical data regarding age of onset and duration of epilepsy, seizure types, medical treatments and outcome was retrieved from case records. Patients underwent a comprehensive set of standard neuropsychometric tests pre-operatively. This battery has been described previously [5] and further detailed in supplemental methods. Data were available for 43 patients and patients were binarized into “decline” or “stable” groups based on their pre-surgical verbal cognitive abilities. A discrepancy of 10 or more between the estimated pre-morbid intelligence and VIQ/VCI was considered clinically relevant cognitive decline [47].

### Statistical analysis

Statistical analysis was conducted using SPSS (IBM corporation, Version 29) and Graph Pad Prism software (Supplemental methods). Non-parametric tests were used for comparison of pathology variables between groups and Wilcoxon signed rank test for differences between superficial and deep white matter. For RNA analysis, Benjamini–Hochberg adjusted p values were presented as shown, otherwise unadjusted p values were noted; *p* < 0.05 was considered statistically significant. Pathway analyses were performed based on Gene Set analysis score, taking to account t-statistics and plots were generated from pathways scores to show variations between groups. Logistic regression analysis was used for comparison of pathology variables between neuropsychology groups using SPSS for multiple imputation of any missing pathology variables. Univariate linear regression analysis was conducted for comparison between DWI and pathology Z scores, with significance taken at *p* < 0.05.

## Results

### Comparisons between epilepsy and control groups

#### White matter myelination and glia

**Fig. 1:**
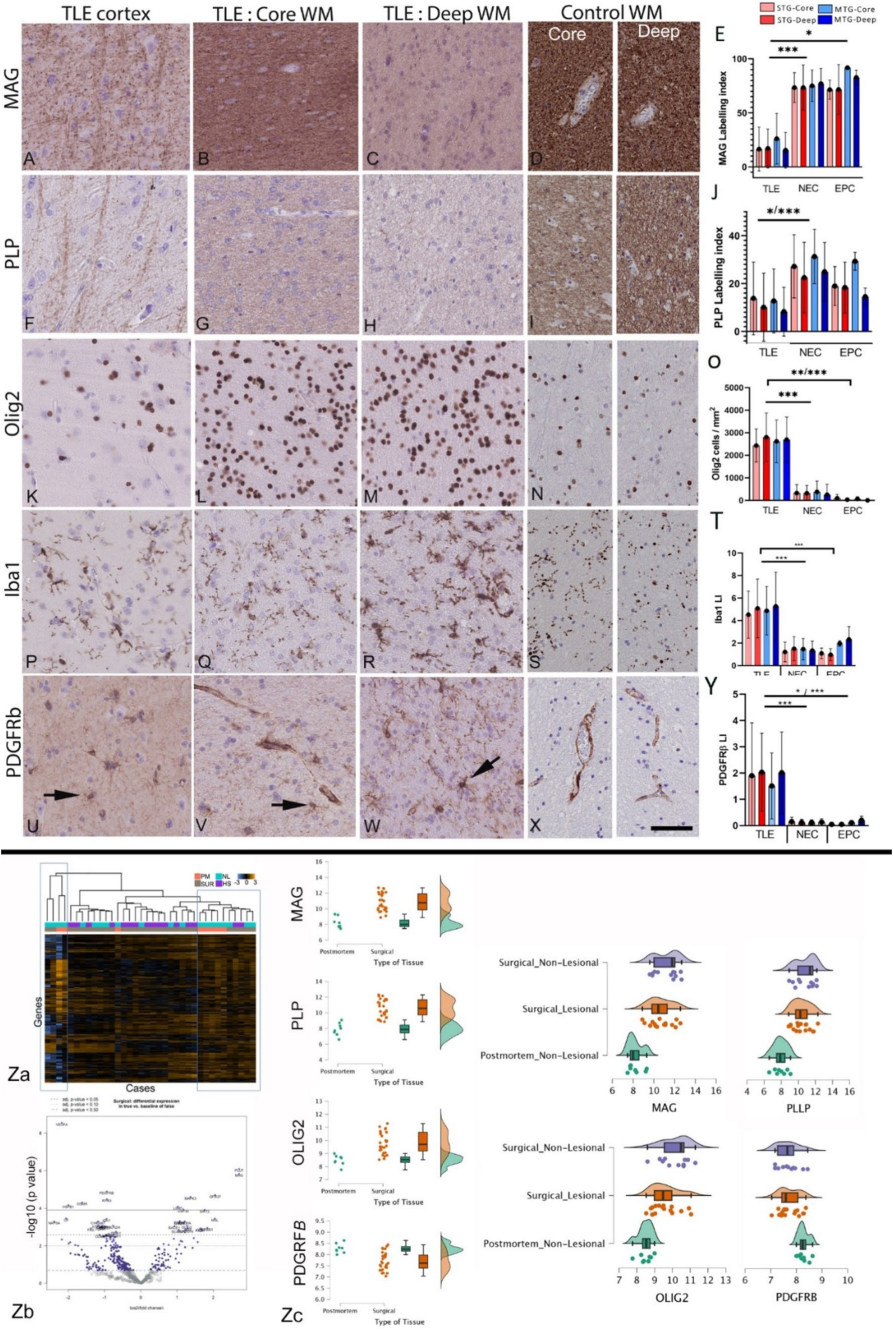
White matter myelin and glial marker analysis in temporal lobe epilepsy (TLE) compared to controls. Top of figure: Pathology quantitation (A-Y): (A). Myelin associated glycoprotein (MAG) in the TLE cortex labelled radial fibers and (B) diffuse, intense axonal labelling in the core white matter with (C) more patchy labeling noted in deep white matter (arrows) (D). In non-epilepsy control post-mortem (PM) cases, white matter showed more uniform and intense labelling (shown for each marker in the core (left) and deep white matter). (E). Bar graph of quantitative analysis of MAG with significantly lower labeling index (LI) in all four regions of interest (ROI) (superior temporal gyrus (STG) core and deep and middle temporal gyrus (MTG) core and deep) in TLE compared to non-epilepsy controls (NEC) and epilepsy controls (EPC). (F). Phospho-lipid protein-1 (PLP) similarly labelled cortical axons and (G) core white matter with (H) weaker labelling in deep but (I) more uniform labelling noted in PM control white matter. (J). Bar graph showed significantly lower labelling in ROI in TLE compared to NEC cases. (K). Olig2 in the cortex with diffuse white matter oligodendrogliosis in TLE cases in (L) core and (M) deep white matter, with evidence of greater densities than (N) control PM white matter (O); This was confirmed on quantitative analysis of Olig2 cell densities with significantly higher values in TLE than NEC and EPC control groups, apart from the deep white matter of the MTG in EPC. (P). Iba1 labelling of ramified microglia in the cortex compared to (Q) core and (R) deep white matter, which both appeared greater than (S) PM control white matter qualitatively and confirmed on quantitative analysis (T) of Iba1 labelling index in all ROI which was significantly higher in TLE than controls, apart from the deep white matter of the MTG in EPC. (U) PDGFRβ in TLE labelled scattered multipolar-glial cells in the cortex and also (V) in the core white matter (arrows) in addition to perivascular cells. (W) In the deep white matter in TLE cases, there was an impression of increased overall labelling (arrow small glial cells) but (X) an impression of fewer PDGFRβ-positive glial cells in PM controls. (Y) The labelling index for PDGFRβ was higher in TLE than NEC and EPC in all ROI. Bar in X is equivalent to 50 microns approx. (redrawn scale bar from ndpi). In the bar graphs, straight lines indicate that all four ROI in the group were significantly different using non-parametric tests (Mann Whitney test). Bars with corners where < 4 ROI showed significance. * p < 0.05, ** p < 0.01, *** p < 0.001. Bottom of figure: Nanostring gene expression analysis of deep white matter (Za-c). (Za). Heat map of gene expression data in all groups with blue lines highlighting clustering of mainly non-lesional cases. (Zb) Volcano plot of significantly upregulated and downregulated genes in TLE surgical cases compared to PM control groups; 135 genes showed at least one-fold significant difference between postmortem and surgical cases. (Zc) Scatter and box plots showing the distribution of the data set, including the median, interquartile ranges, minimum and maximum, for specific genes (MAG, PLLP, OLIG2 and PDGFRβ) and expression in surgical lesional, non-lesional and PM control groups: MAG, PLLP and Olig2 showed significantly reduced mRNA expression in postmortem cases compared with surgical cases (p < 0.001), whereas PDGFRβ revealed higher gene expression in postmortem cases compared with surgical cases (p < 0.001)

On qualitative analysis of TLE cases, MAG showed more intense labelling of myelinated axons in the gyral core and cortical radial fibres than the deep white matter (Fig. 1A–C) whereas more uniform labelling was noted in control PM white matter (Fig. 1D). PLP findings were similar to MAG but with an overall reduced intensity (Fig. 1F–I). Relatively uniform patterns of axonal labelling were noted in both core and deep white matter in TLE with neurofilament markers (NF-L, NF-M and NF-L) (Supplemental Fig. 5C–E). Olig2 showed diffuse increase in white matter oligodendroglia in TLE (Fig. 1K–N) and Iba1, increased white matter microglia relative to cortex and controls (Fig. 1P–S). PDGFRβ highlighted pericytes in relation to vessels in addition to scattered single multipolar-glial cells through the white matter and cortex (Fig. 1U–W). In TLE cases, there was a clear gradient, with increased numbers in deep compared to gyral core white matter and qualitatively fewer PDGFRβ-positive glial cells in controls (Fig. 1X). NeuN labelled single interstitial neurons in both core and deep white matter in TLE and control groups (Supplemental Fig. 5A). Tenascin C showed predominant expression in the white matter matrix, extending into the deep cortex in some areas around blood vessels and glial cells (Supplemental Fig. 5B).

Quantitation of MAG was significantly lower in TLE than NEC and EPC in STG and MTG core and deep white matter ROIs (*p* < 0.001 and *p* < 0.05); PLP was significantly lower in TLE than NEC in these regions (*p* < 0.05) but not EPC (Fig. 1E, J, Suppl Table 2). In contrast, Olig2 density, PDGFRβ and Iba1 LI, were all significantly higher in white matter ROI in TLE than controls (*p* < 0.001) but with greater differences noted between TLE and NEC than EPC groups (Fig. 1O,T,Y, Suppl Table 2).

#### White matter microangiopathy

**Fig. 2:**
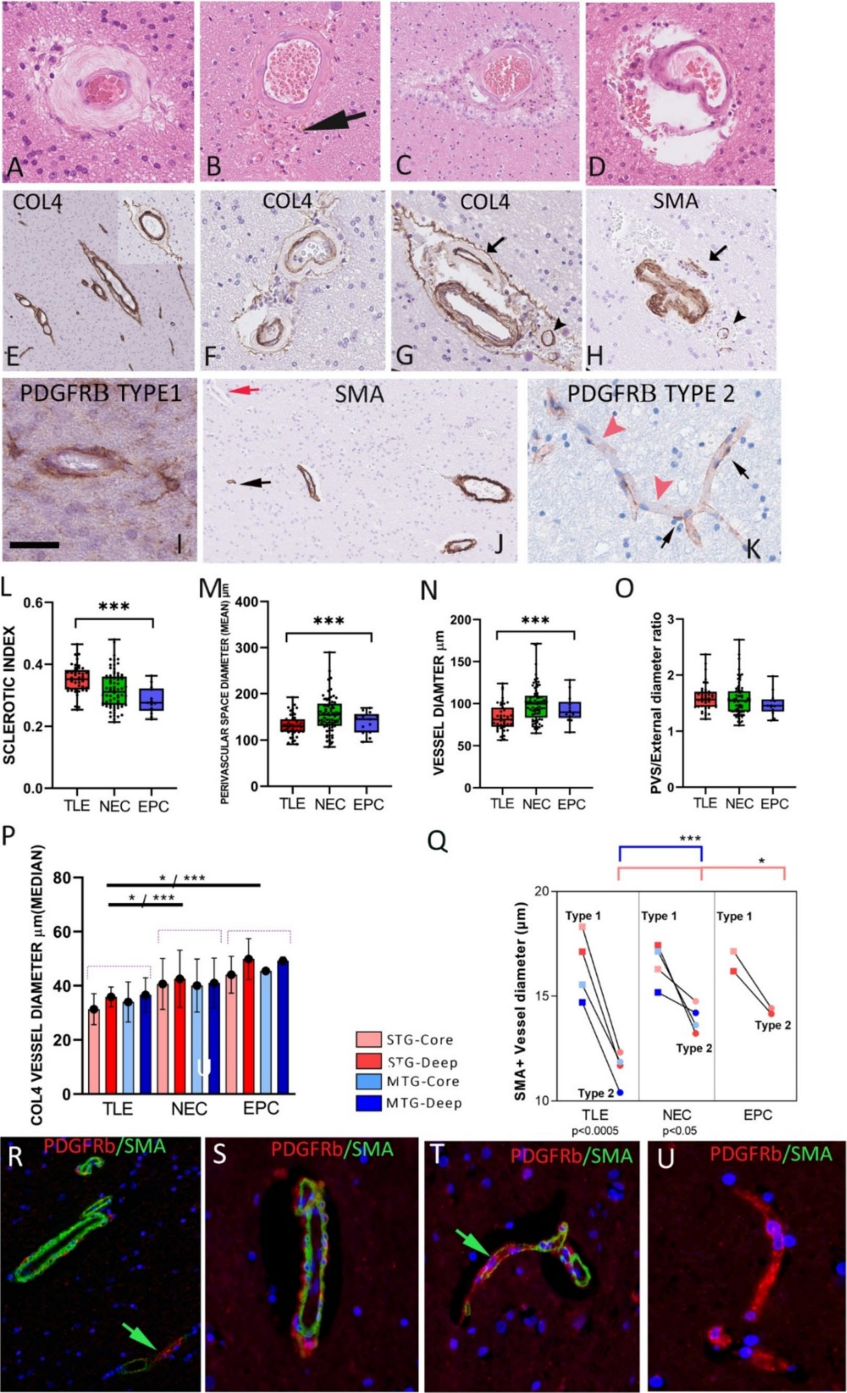
White matter vascular marker analysis in temporal lobe epilepsy (TLE) compared to controls. The spectrum of white matter small vessel disease (SVD) in TLE with (A) marked arteriolosclerosis with hyaline thickening of the media on H&E stain, (B) perivascular pigment laden macrophages, (C) perivascular space (PVS) dilatation and prominent corpora amylacea and (D) Perivascular space dilatation without vascular sclerosis. (E) COL4 labelling of white matter vessels of all calibre; inset showing an observation noted in some TLE cases of splitting of the COL4 layer with intervening PVS. (F) COL4 labelling of a small vessel with sclerosis and in (G) with splitting or ‘dissection’ of COL4 layer. (G) and (H) show the same white matter vessels in serial sections, labelled with COL4 and SMA (arrow and arrowheads); there is sclerosis of one vessel and splitting of COL4 labelling which is not observed on SMA, although loss of SMA + cells in the media is noted in the hyalinized vessels. (I) PDGFRβ labelling of vessels with complete circumferential pericyte labelling (Type 1 vessel). (J) SMA labelling of perivascular smooth muscle cells in arterioles in addition to pericytes around small capillaries which were SMA positive (black arrow) or negative (red arrow). (K) Intermittent labelling of PDGFRβ around capillary channels (Type 2 vessel). (L) Box and whisker plot (showing quartiles and maximum and minimum values) of sclerotic index in TLE cases compared to non-epilepsy control (NEC) and epilepsy control (EPC) groups. Significantly greater vascular sclerosis was noted in the TLE group. (M) Perivascular space (PVS) diameter, (N) vessel diameter and (O) the ratio of PVS to external vessel diameter are shown with lower PVS and diameter in the TLE group (Kruskal–Wallis test). (P) Bar graph of mean values of COL4 vascular diameter (microns) (and standard deviation in error bars) for all ROI in TLE, NEC and EPC groups with smaller mean values in TLE. (Q) Line graphs of SMA vascular diameters (microns) for Type 1 and Type 2 vessels in different ROIs in TLE, NEC and EPC control groups. Group differences (Mann–Whitney tests) are shown (top) and paired differences between type 1 and 2 vessel diameters within groups (Wilcoxon signed rank test, bottom), with greater significance noted in the TLE group which showed smaller mean size of type 2 vessels. (R-U) Double labelling (SMA/PDGFRβ) of TLE cases and PM controls (S,T) confirming co-labelling of small arterioles in white matter (R,S) as well as focal co-localisation of labelling in some small capillaries (green arrows (R,T)). However, many small capillaries were devoid of SMA labelling (U). For graphs * p < 0.05, ** p < 0.01, *** p < 0.001. Bar is equivalent to approx. 20 microns in A-D, 50 microns in G, H, R-U, 35 microns in I, K, 150 microns in E, J (Bar redrawn from original npdi scales)

In some TLE cases, scattered medium sized white matter vessels showed variable degrees of hyaline thickening of the vessel wall (vascular sclerosis/SVD) on H&E (Fig. 2A) although this was not evident in all vessels or cases. PVS expansion, with pigment laden macrophages and corpora amylacea was noted around some vessels, including those without sclerosis (Fig. 2B–D). COL4 + highlighted the basal lamina of white matter vessels of all calibre (Supplemental Fig. 1C). Frequent findings were ‘splitting’ of the COL4 + layer to produce a double-layer, particularly in vessels with expanded perivascular spaces and sclerosis (Fig. 2E-G). This microvascular dissection was not appreciated with PDGFRβ or SMA (Fig. 2H). PDGFRβ (Fig. 2I, K) and SMA (Fig. 2H, J) labelling was noted in arterioles and also the smallest capillaries. Vessels showed continuous circumferential labelling with SMA or PDGFRβ (termed Type 1) or discontinuous labelling (Type 2) in both TLE and controls (Fig. 2I, K and Supplemental Fig. 4D, E). Double labelling with PDGFRβ/SMA in selected cases and controls confirmed co-localisation of labelling in some arterioles and capillaries (Fig. 2R–U), however, many smaller capillaries were PDGFRβ + /SMA- (Fig. 2U), whereas PDGFRβ-/SMA + vessels were not observed in either TLE or controls.

#### Vascular sclerotic index

In cerebrovascular disease, an SI value of greater than 0.3 represents mild, and greater than 0.5 severe SVD [10]. The mean SI was 0.35 in TLE and significantly higher than controls (NEC 0.31, EPC 0.28; *p* < 0.001, Fig. 2L). The SI was not higher in two TLE patients with identified cerebrovascular disease risk factors (hypertension and/or Type 1 diabetes) than those without. Mean vessel diameters and PVS were lower in TLE than control groups but not the ratio between these values (Fig. 2M–O).

#### Vascular size, types, and densities

The median diameter of COL4 vessels was lower in TLE across all white matter ROI compared to control groups (*p* < 0.05–0.0001, Supp Table 2, Fig. 2P). COL4 and SMA vascular densities were both significantly higher in TLE than NEC in the MTG core but not other ROI (*p* < 0.005, *p* < 0.05, Mann Whitney test) (Supplemental Fig. 6 A,B, Suppl Table 2). Classification of different vessel sizes quantified on PDGFRβ, showed greater differences in the deep than core regions in TLE than NEC, including higher capillary and arteriole densities in the MTGD (Mann Whitney, *p* < 0.05, Supplemental Fig. 6C). These findings support reduced mean size of small vessels in TLE, but without a uniform increase in small vessel densities across white matter ROI.

#### Pericyte distribution

SMA + and PDGFRβ + vessels were classified as type 1 or 2 based on complete or incomplete vascular coverage, respectively. In TLE, PDGFRβ type 1 vessel densities were significantly greater, whereas type 2 PDGFRβ vessel densities were lower in deep white matter ROI in TLE compared to controls (*p* = 0.015–0.009) (Supplemental Fig. 6D,F, Supplemental Table 2); significant differences were not observed with SMA, however (Supplemental Fig. 6H,J). There was evidence that Type 2 vessel were smaller in TLE than control groups in some ROI (SMA STGC p < 0.05; SMA MTGD *p* < 0.001; PDGFRβ STGD *p* < 0.05) whereas type 1 vessel diameters were larger than controls (PDGFRβ MTGC *p* = 0.04, STGD *p* = 0.04, MTGD *p* = 0.001) (Supplemental Fig. 6 E,G,I,K). Paired tests showed significantly greater difference between type 1 and 2 vessels size with SMA in TLE cases than controls (*p* < 0.001 all ROI, Fig. 2Q). Analysis of double labelling for SMA and PDGFRβ showed higher densities of PDGFRβ + /SMA + and PDGFRβ + /SMA- in TLE than controls, reaching significance for type 2 vessels in the deep white matter (*p* = 0.028, Figure Supplemental Fig. 6I). These findings support increased pericyte vascular coverage of small vessels as well as altered relative expression of PDGFRβ and SMA in capillary pericytes in TLE.

#### Comparisons of gyral core to deep white matter

**Fig. 3:**
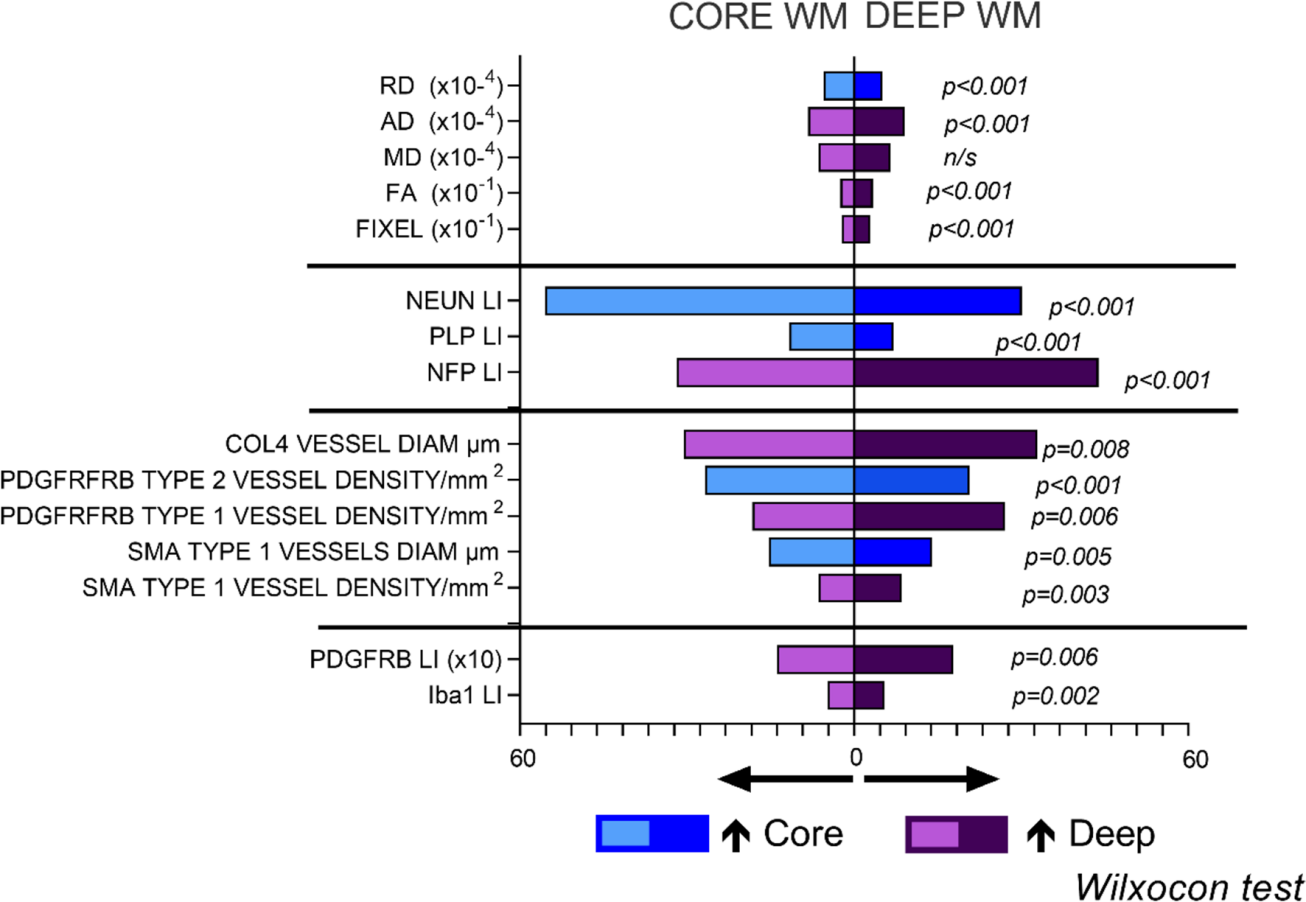
Comparison of deep and superficial white matter pathology and DWI variables in TLE. Pyramid graphical representation of DWI measures (top) and pathology factors which showed significant differences between the superficial and deep white matter (data shown is average scores across all core and deep white matter regions (superior, middle and inferior temporal gyri) and p values shown with Wilcoxon rank score test). AD = Axial Diffusivity, MD = Mean Diffusivity, RD = Radial Diffusivity, FA = Fractional Anisotropy, FIXEL = Fixel Based Analysis Measurement

We further explored differences between gyral core and deep white matter for pathology variables, comparing average values across all gyri in TLE (Fig. 3) and control groups (Supplemental Fig. 7A,B). PLP LI was higher in the core than deep WM in TLE (*p* < 0.001) and controls (*p* < 0.05). For glial cells both Iba1 and PDGFRβ LI were increased in the deep white matter in TLE (*p* < 0.01) but no differences noted in controls whereas OLIG2 showed a core > deep gradient in control groups only (*p* < 0.05). For small vessels, notable findings were that type 1 vessel densities were higher in the deep than core white matter (for SMA and PDGFRβ (*p* < 0.01)) with an opposite gradient noted in controls for PDGFRβ (*p* < 0.05). In contrast PDGFRβ type 2 vessel densities were higher in the core white matter in TLE (*p* < 0.001) with no differences in controls. These observations highlight a superficial to deep white matter gradients for glio-vascular pathology in TLE.

Within both core and deep white matter, correlations between the vascular morphometric measures and glial/myelination were observed in TLE (Supplemental Fig. 7C,D). For example, SI positively correlated with PDGFRB glia and negatively with axonal neurofilament in the deep white matter. PVS negatively correlated with core PLP. Further correlations between glial, microglia, matrix protein Tenascin-C and small vessel densities suggest interactions in TLE.

#### RNA expression data in TLE

Epilepsy surgical and non-lesional postmortem cases showed clustering of cases into three groups with one group enriched with surgical cases (Fig. 1Za). 135 genes showed at least one-fold significant difference between postmortem and surgical cases (unadjusted *p* < 0.05, 40 genes when using Benjamini–Hochberg adjusted *p* value, *p* < 0.05, Fig. 1Zb), and bioinformatic analysis using Reactome revealed that these genes were associated with Developmental Biology and Signaling Transduction pathways including EGR2 and SOX10-mediated initiation of myelination and signaling by VEGF. Genes of interest associated with myelination and oligodendroglia including MAG, PLLP (proteolipid plasmolipin) and Olig2 showed significantly reduced mRNA expression in postmortem cases compared with surgical cases (*p* < 0.001), whereas PDGFRβ revealed higher gene expression in postmortem cases compared with surgical cases (*p* < 0.001) (Fig. 1Zc).

#### Pathology and RNA expression in HS cases

Most cases had HS (33/44), but we did not observe significant differences between mean core and deep white matter pathology variables in the fewer cases without HS apart from PDGFRβ type 1 vessels which had increased diameter and reduced PLP in core regions in HS (*p* ≤ 0.05) (Supplemental Fig. 8A). Gene expression analysis, however, showed more significant findings with reduced MAG, PLLP and Olig2 in HS compared to non-lesional epilepsy surgical cases (p < 0.05, Supplemental Fig. 8B,C) in addition to genes known to interact with MAG such as MBP, SOX10 (*p* < 0.05) and NGFR (*p* < 0.05) according to STRING database [69]. MAG expression and genes in myelination pathway positively associated with genes in the angiogenesis and activated microglia and cytokines pathways but with greater correlation in non-HS cases (Supplemental Fig. 8D-H). These findings support evidence for a relatively greater reduction of myelination in HS/TLE and interaction of myelination, angiogenesis pathways and neuroinflammation in non-HS cases.

#### DWI metrics in relation to white matter pathology

**Fig. 4:**
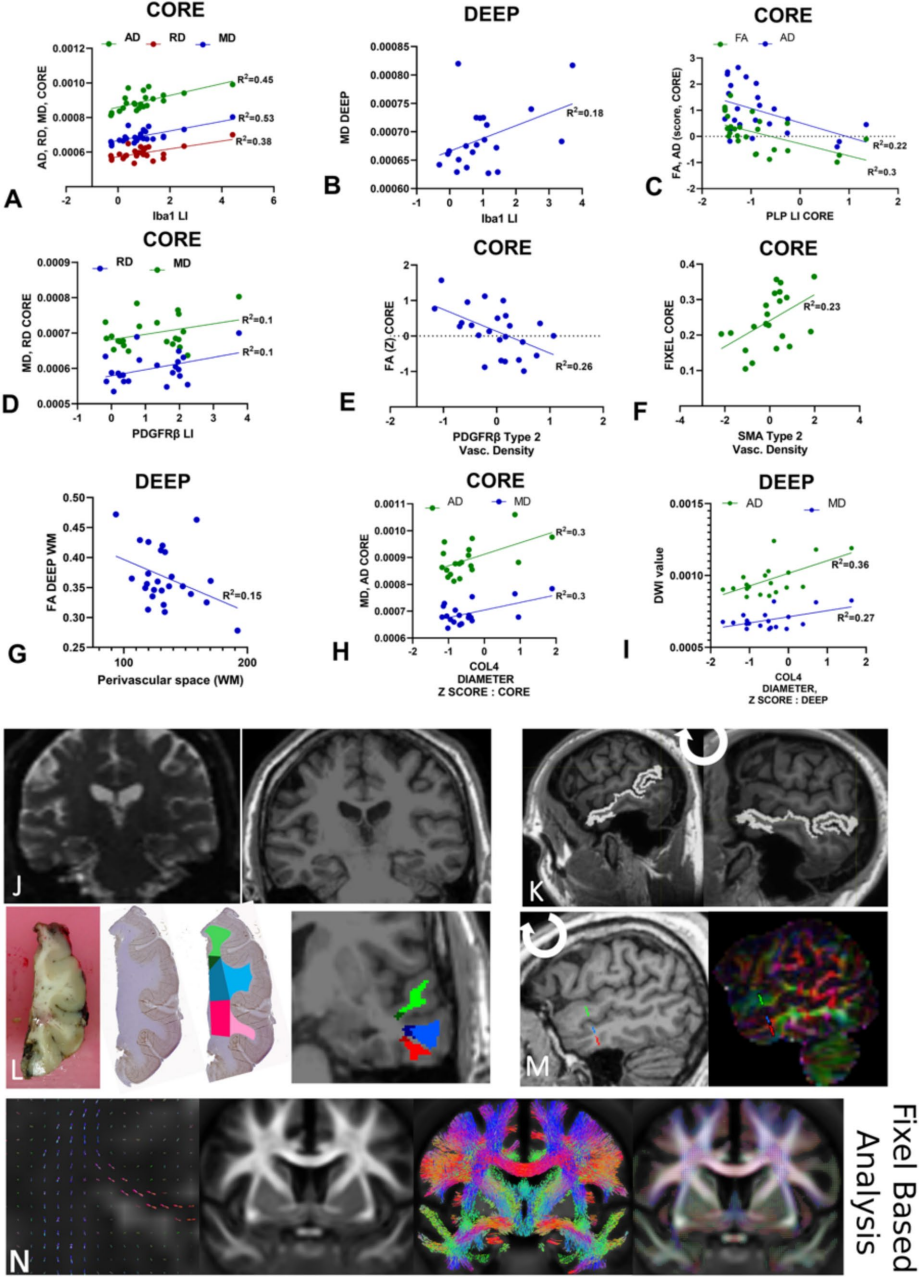
Diffusion weighted MRI (DWI) and Fixel-based analysis (FBA) with pathology correlations. Linear regression analysis of DWI measures averaged across either all core or deep white matter regions (also regressed using Z scores relative to control DWI data, where shown) against pathology variables in same regions (as mean scores and Z scores compared to control cases); data is shown where significant regressions were identified on univariate analysis (See also supplemental Table 3): (A) Iba labelling and AD, MD, RD (core white matter), (B) Iba1 and MD (deep), (C) Phospholipid protein (PLP) and FA, AD (core), (D), PDGFRβ Labelling index (LI) and MD, RD (core), (E) PDGFRβ type 2 vascular density and FA (core), (F) SMA type 2 vascular density and Fixel (Core), (G) perivascular space measurement (PVS) and FA (Deep) (H) COL4 vascular diameter and AD and MD (Core) (I) COL4 vascular diameter and AD and MD (Deep). These confirmed the main associations for DWI and FBA with pathology were in the core white matter and for vascular pathology measures (see text for details). Pipeline for the DWI analysis: (J) Diffusion images following denoising, un-ringing, motion, eddy and field bias corrections were aligned to T1 and diffusions interpolated to match voxel size of T1 (1 mm). (K) Middle temporal gyrus (MTG) was rotated to align flat to enable orientation with brain slice, (L) Regions of interest (ROI) in white matter (superior temporal gyrus green shades, middle temporal gyrus blue shades, inferior temporal gyrus red shades with deeper colour shade representing deep ROI) were best matched and co-registered on MRI and pathology histological sections using Freesurfer white matter parcellation to manually segment gyri into the core and deep white matter (note the histology sections shown for illustration only or MRI and not aligned to MRI slice axis shown). (M) MRI images were then rotated back to extract diffusion tensors for each region. (N) Fixed-based-analysis (Left to right): 1. Individual orientation distribution function (ODF) were calculated, 2. registered to create a healthy population template, 3. which was used to create a SIFT filtered tractogram, for 4. Fixel-based analysis. RD = Radial Diffusivity, AD = Axial Diffusivity, MD = Mean Diffusivity, FA = Fractional Anisotropy, FIXEL = Fixel Based Analysis Measurement, LI = labelling index

**Fig. 5:**
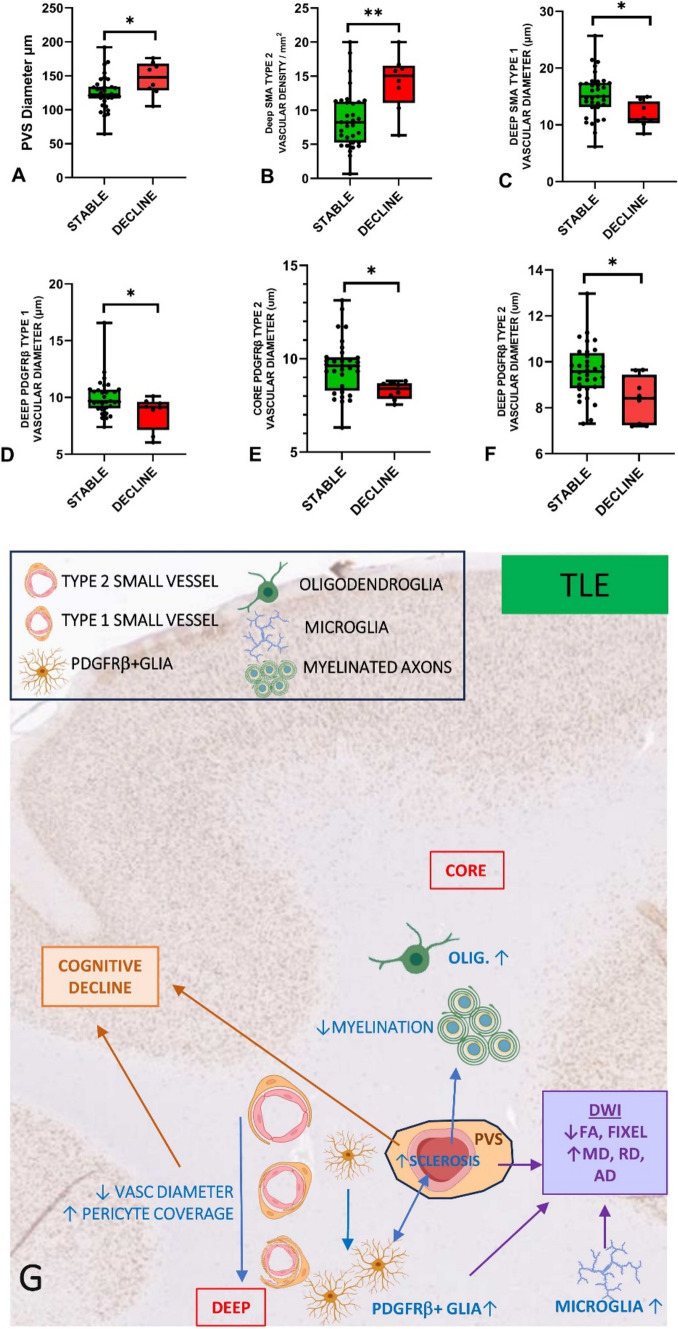
Correlations with microvascular pathology and neuropsychology. (A-F). Logistic regression analysis of pathology measures in groups with and without pre-operative verbal cognitive decline (see also Supplemental Table 4) showing significant differences in vascular pathology measures in the decline group, represented graphically as box plots (median, 25th and 75th centile, range): (A) Perivascular space (PVS) and (B) SMA type 2 vascular density were increased in the decline group whereas (C) SMA type 1 vessel diameter (deep) (D) PDGFRβ type 1 vessel diameter (deep), (E) PDGFRβ type 2 vessel diameter in core and (F) deep white matter were significantly lower in the decline group. (G). Summary diagram based on all observations of regional alterations and relationship of small vessels measurements with other cellular components in TLE white matter (blue text and arrows) and which factors might impact on DWI changes (purple arrows) and cognition (brown). In brief, temporal lobe epilepsy is associated with both increased arteriolosclerosis and vascular pericytes; the later may represent a protective adaptation. Increased parenchymal PDGFRβ cells, which correlate with degenerative vascular sclerosis, are increased particularly in the deep white matter. Epilepsy is associated with white matter oligodendrogliosis and microglial increase, the latter particularly in deep regions. Increased perivascular space and reduced small vessel size are linked to cognitive decline. DWI alterations in the core are mainly influenced by increased glia density, PVS and small vessel changes. Icons created with Biorender

DWI measures showed regional differences in TLE with RD values higher in the gyral cores than deep white matter whereas AD, MD, FA and FDC were higher in the deep regions, using data from superior, middle and inferior temporal gyri (Fig. 3). Linear regression analysis of DWI values with pathology variables revealed greater significance in core than deep white matter regions (Supplemental Table 3). Glial labelling (Iba1 and PDGFRβ) increased with higher diffusion parameters (AD, RD and MD, *p* < 0.05 to < 0.0001) and higher myelin (PLP) labelling with lower AD (p < 0.05) and FA values in the core (Fig. 4A-D). Regarding vasculature, type 2 vessel density regressed with FA and FDC measures in the core white matter: higher PDGFRβ and SMA with lower FA and greater FDC, respectively (*p* < 0.05), Fig. 4E,F). In the deep white matter, greater mean PVS associated with lower FA (Fig. 4G). In addition COL4 vessel diameters positively correlated with MD and AD in both core and deep white matter (Fig. 4H,I). These observations suggest that although DWI differences noted between core and deep white matter may partly be explained by anatomical differences in axon bundle organisation, alterations of glial density and vascular structures in TLE further influence diffusion measures (Fig. 5G). In the ten cases with paired DWI and gene-expression data from deep white matter, positive association between myelination genes (MOG, MAG, MBP, PLLP) and FA and negative relationship with RD was noted (Supplemental Table 3).

#### White matter pathology and neuropsychometry

Nine of 43 patients were classified as having verbal decline in cognitive function pre-operatively and 9/43 had impaired working memory at the time of surgery. Using logistic regression analysis for mean deep and core white matter values for groups with or without cognitive decline, we found higher PVS measure (Fig. 5A) and SMA type 2 vessel densities (Fig. 5B) in the decline group, as well as reduced vascular diameters for deep type 1 vessels (SMA and PDGFRβ) and type 2 vessels in core and deep (PDGFRβ) (Fig. 5C–F). There was also a trend for lower MAG LI (Supplemental Table 4). There were no significant relationships for pathology variables and working memory impairment at the time of surgery. These findings support a relationship between microvascular alterations, including vessel calibre in those patients with TLE who have declined in their general verbal intellect over time prior to surgery.

#### White matter pathology and epilepsy history

A positive correlation between age and SI was noted in the post-mortem controls only (Spearman’s *r* = 0.594). The non-epilepsy cases were significantly older than the TLE cases (*p* < 0.001). There was no correlation between SI or PVS and duration of epilepsy or seizure types in the TLE group but small vessel measurements were more abnormal the longer the duration of epilepsy, including increased vascular diameter (SMA, *p* < 0.05) and reduced vascular density (COL4, *p* < 0.05) in the core white matter (Supplemental Fig. 9A,D). Regarding vessel types, type 1 vessel densities increased with age whereas type 2 vessels decreased with duration of epilepsy (PDGFRβ, Deep; *p* < 0.05) (Supplemental Fig. 9C,F). In addition, reduced axonal labelling was observed with age (NF-M, Deep; *p* < 0.05) and more pronounced the longer the epilepsy (NF-H, Deep; *p* < 0.01) (Supplemental Fig. 9B,E). There were no differences for vascular, glial, axonal or myelin markers in relation to hemisphere side; 23/45 patients were categorized as seizure-free following surgery at 2 years follow up [80], but there was no statistical relationship between seizure outcome and pathology measures. Gene expression of MAG, PLLP, Olig2 and PDGFRβ was compared between patients with an age of surgery lower or equal to 40 years and over 40 years of age; the older cohort showed lower expression of MAG, PLLP, OLIG2 mRNA whereas PDGFRβ RNA level appeared to be slightly higher (Supplemental Fig. 9G). These findings suggest that longer duration of epilepsy is associated with age-related alterations of myelin and microvasculature changes.

## Discussion

White matter vessel disease in TLE is of relevance through its impact on brain perfusion, myelin integrity and ultimately, cognitive function [51, 55]. In this morphometric and transcriptomic study, we identified alterations to white matter vessels, including degenerative sclerosis and altered distribution of vascular pericytes compared to controls with differences noted between core and deep white matter. Vascular changes correlated with MRI measures of white matter integrity (DWI and FBA), duration of epilepsy and cognitive decline, supporting dynamic changes of clinical importance.

### Small vessel degenerative microangiopathy in TLE

There is growing evidence that microvascular dysfunction occurs in epilepsy and is detrimental to both seizure control and its comorbidities [51, 55] but has been under investigated in surgical series. An early study showed that SVD characterized by increased wall thickness of capillaries occurred in spiking areas [40] and a ‘spongiform angiopathy’ of the white matter was reported in young patients, with spitting of the vascular basement membrane and enlargement of the PVS [27]. In our series of adult TLE cases, we identified a mild increase in small vessel hyaline arteriolosclerosis and an elevated mean sclerotic index, a standard morphometric measure used for degenerative SVD in aging and dementia [7]. This was compared to control groups, which included both non-epilepsy patients (with a significantly older mean age) and non-TLE epilepsy controls. We included an epilepsy control group without focal brain lesions or TLE to identify any epilepsy-related pathology, and for some markers, as PLP and glial markers, measurements were intermediate between TLE and non-epilepsy cases. A relationship between age and sclerotic index was lacking in the TLE group, suggesting age-independent mechanisms for the SVD. Subtypes of brain arteriolosclerosis with different causes, including genetic, metabolic and hemodynamics are recognised [7]. It is plausible that repetitive vascular dysfunction, including BBB disturbances following seizures [61], is a contributing factor in TLE and inter-ictal BBB dysfunction recently correlated with memory dysfunction [56].

We noted splitting of the basement membrane on COL4 stain in TLE cases and prominent PVS, the latter shown to be a predictor of cognitive impairment in vascular dementia [7, 30]. In addition, SI and increased PVS correlated with reduced white matter myelin and axonal labelling which may imply tissue perfusion impairment. PVS enlargement also correlated with pre-operative cognitive decline as well as reduced white matter FA on DWI, indicating clinical and imaging relevance of this degenerative feature. We did not, however, observe increased PVS compared to post-mortem controls, which may be explained by agonal retardation of interstitial fluid clearance. The PVS is part of the CNS glymphatic system [79] and failure of this system for metabolite clearance is implicated in neurodegeneration [76]. Accumulation of perivascular corpora amylacea (also called ‘wasteosomes’) and pigment-laden macrophages is a common observation in TLE [58] as in the present series, in support of impaired periarterial drainage. Recent studies also showed ipsilateral glymphatic dysfunction in TLE with DTI-ALPS imaging [86] warranting further in-depth study of these processes in relation to clinical and cognitive impairment in epilepsy.

### Small vessel regenerative microangiopathy in TLE

In the present study, white matter RNA expression analysis showed an increase in angiogenesis pathways. Using COL4, SMA and PDGFRB vascular markers, there was some evidence to support a quantitative increase in vascular density in TLE cases, although noting this was neither uniform nor a consistent pattern through white matter regions. Furthermore, as it was not feasible to calculate the absolute number of vessels, we cannot exclude that our quantitative data partly reflect regional reduced white matter volume than a real increase in small vessel number. There is controversy if angiogenesis occurs in TLE-HS. Increased vessel number was reported in the hippocampus in TLE patients [59] but not in other studies [2] and there is limited data on cortical or white vasculature (recently reviewed in [73]). Enhanced angiogenesis was shown using proteomic analysis of epileptogenic cortex [32] and quantification of vessels using CD34 endothelial marker in a range of focal malformations noted a correlation between grey matter vascular density and epilepsy duration, but not in white matter [75].

We noted more consistent evidence for a reduction in small vessel size in TLE white matter with all markers (COL4, SMA and PDGRRβ) and increased pericyte coverage. Vascular caliber and contractility are relevant to white matter perfusion and dependent on contractile vascular smooth muscle cells and pericytes. Higher pericyte to endothelial ratios occur in the cerebral vasculature, considered essential for BBB integrity and to regulate local blood flow for neurovascular coupling [1]. There is experimental evidence of dynamic changes to pericytes in epilepsy; pericytes show microscopic vasocontractions following seizures in animal models [37]. In spiking areas in surgical samples, degeneration of pericytes was noted compared to non-spiking regions [40]. Following status epilepticus in models, initial loss of perivascular cells was followed by an increased turnover with redistribution of PDGFRβ + along vessels [3, 43]. In slice cultures, pericyte injury following induced seizures was associated with neurovascular decoupling, BBB impairment and irreversible vascular constriction [51] and interestingly smaller vessel diameters was observed with increased pericyte number [3].

Morphological diversity of pericytes is recognized; ‘ensheathing’ pericytes cover 95% of the endothelial surface in first branch capillaries, whereas mesh and strand pericytes on downstream capillaries have less complete coverage [22, 65, 85]. Ensheathing pericytes between arterioles and first branch capillaries were shown to strongly express SMA and act as pre-capillary sphincters, regulating local capillary blood flow [85] whereas there is reduced SMA gene expression in distal mesh and strand pericytes [22]. In the current study we classified type 1 or 2 vessels based on complete or incomplete circumferential coverage of pericytes as we were quantifying on thin sections with vessels in random directions. We observed increased type 1 and reduced type 2 vessels, particularly in the deep white matter in TLE than controls with PDGFRβ, in support of increased pericyte vessel coverage. In addition, SMA was noted in smaller vessels compared to controls, supporting increased capillary contractile function. Pericyte vessel coverage was increased with longer duration of epilepsy, consistent with this being an acquired dynamic process. One interpretation of the altered relative expression of SMA to PDGFRβ in pericytes together with overall smaller vessel coverage, is a functional alteration of contractility which could impact on haemodynamic regulation and white matter perfusion [22]. Our observation is the opposite to findings in SVD associated with aging and dementia where loss and degeneration of pericytes occurs (reviewed in [71]). In addition, we also noted a relationship between reduced vascular size and cognitive impairment supporting the potential relevance of this TLE-related SVD warranting further investigation in larger cohorts.

### Diffusion MRI and superficial white matter vascular pathology

DWI alterations are common in TLE [25] although few studies report pathology correlations [12, 21, 41]. Reduced FA and increased MD in the temporal lobe and pole in TLE [39, 81] has been interpreted to reflect degradation of myelin sheaths, reduced axonal number, increased extra-axonal space and reactive astrogliosis [9, 26, 81]. Small vessel disease in white matter may result in diffuse changes to FA and MD in epilepsy (Wang, Zuo et al. 2024) but there have not been focused investigation in TLE. Previous DWI studies in TLE have also reported specific superficial white matter abnormalities [39, 72] related to network dysfunction, memory impairment [83] and cognitive phenotype [57].

We noted more frequent correlations between diffusivity in the core white matter and, furthermore, with vascular size and glial density (PDGFRβ and Iba1) than myelin markers. An inverse correlation between FA and PLP in the core may reflect a preferential dysmyelination in projection white matter fibres. FBA is a recent DWI tool that renders a measure of both white matter fibre distribution and orientation in addition to density [13] and has been noted to discriminate white matter alterations in TLE from other epilepsies [45]. Such methods are relevant to analysis of superficial temporal lobe white matter with different directionality of crossing ‘U’ and radial projection fibres, and reflected in the gradient we observed in DWI measures between core and deep compartments. Correlations between FA/FIXEL and vascular density and PVS support that degenerative vascular disease can impact on diffusion metrics. This highlights the influence of vascular pathology, through either direct or its indirect effects on other white matter structures, to diffusion imaging.

The increased parenchymal PDGFRβ cells in TLE were a striking finding and previously reported in focal epilepsy [20, 43, 63]; they may represent a NG-2 glial progenitor type. Their biological roles as a source of new pericytes and contribution to CNS scarring and vascular sclerosis is of ongoing interest [28, 34, 54] and we noted a correlation with vascular sclerosis in this cohort. Of note, despite immunohistochemical evidence of increased parenchymal and perivascular PDGFRβ + cells in TLE, bulk RNA transcripts of white matter showed lower expression than controls. The PDGFRβ signaling pathway is critical to developmental angiogenesis and recent evidence confirms roles in ongoing maintenance of pericytes in the adult brain [74] where a complex process of receptor internalization and signal attenuation is shown to fine tune PDGFR activity and responses [60]. Such sequestration may explain the discordance in our findings between gene and cellular expression in TLE and highlights the need for single cell analysis to explore any alterations in this signaling pathway in epilepsy. Indeed, altered PDGFRβ signaling with loss of pericytes has been identified in AD as a potential therapeutic target [64] and the loss of PDGFRβ + white matter pericytes in post-stroke dementia [14, 18].

### Myelin and oligodendroglia in TLE

Alteration to white matter axonal myelination is recognised in epilepsy with evidence for both a reduction [17, 49] and increase [36]. Differences in myelination dynamics between epilepsies may reflect neuronal and network activity, oligodendrogliosis, neuroinflammation but also tissue hypoperfusion [35]. White matter myelination has been more comprehensively investigated in focal cortical dysplasia [11] with reduced myelin [63] and downregulation of myelin-associated transcripts in the dysplastic region [16], possibly driven by mTOR effects on oligodendroglia [24]. In TLE, a reduction in white matter, projection myelinated axons and increased extra-axonal space has been reported [9, 21, 49]. We also noted greater myelin transcript reduction in HS than non-HS cases and with increasing age at surgery.

Previous quantitative studies of white matter myelination in TLE utilized tinctorial stains or myelin basic protein (MBP) [21, 41]. We studied MAG, which is highly susceptible to reduced tissue oxygenation and PLP a more resistant myelin protein; these comparative markers have been used in post-mortem studies as indirect measures of white matter hypoperfusion in vascular dementia [44] and may reflect dynamic changes to myelin. We noted a reduction of MAG, and to a lesser extent PLP, compared to non-epilepsy controls, particularly in deep white matter in TLE. One interpretation is that a progressive degeneration of projection white matter fibers occurs and episodic tissue hypoperfusion in seizures may be an influencing factor. In contrast, tissue transcripts of white matter myelination genes were increased, which likely reflect the observed oligodendrogliosis. White matter oligodendrogliosis is recognized in focal and experimental epilepsy including TLE [31, 36, 62, 66] although the cause and functional significance remains unclear. Recently, oligodendroglia hyperplasia in frontal lobe epilepsy has been linked with deficient white matter myelination and SLC35A2 brain mosaicism (MOGHE) [8]. Further understanding of any maladaptive or dysfunctional myelination arising in TLE, and vascular versus genetic causes, is needed as it may both exacerbate seizures and impair optimal neurological function [35].

### Limitations of the study

Although we selected control cases with shortest post-mortem intervals and fixation times, we cannot exclude that tissue handling factors between surgical TLE samples and agonal changes influence pathology and vascular measures. In some ROIs, there was missing pathology data due to technical and tissue quality reasons. The subsets with cognitive decline were relatively small in this cohort which was selected primarily on availability of DWI. The patients with TLE were on varied and multiple anti-seizure medications (ASM) over their treatment history (average number of ASMs trialed 7 (range 1–16), Supplemental Table 1); some ASMs including carbamazepine and oxcarbazepine have been linked with an increased risk of vascular disease [48] which may be a factor influencing the arteriosclerosis observed in TLE. We were careful to exclude cases with prior SEEG investigations in the cohort for RNA extraction as well as electrode injury sites in the pathology studies, but we cannot entirely exclude an effect on white matter glial cells in the latter cohort.

## Conclusions

In summary, mild age-independent small vessel arteriolosclerosis coupled with altered distribution of white matter pericytes and increased extension to smallest vessels represent acquired and adaptive alterations in TLE with potential haemodynamic influences on white matter myelination, glial proliferation, MRI diffusion alterations and consequently, might explain cognitive decline. Further investigations in larger cohorts paralleled with other neurodegenerative and cortical pathology markers and genetic risk factors for neurodegenerative and vascular disease is the next step.

## Supplementary information

Below is the link to the electronic supplementary material.

Supplementary file1 (DOCX 77 KB)

Supplementary file2 (DOCX 63 KB)

Supplementary file3 (DOCX 37 KB)

Supplementary file4 (DOCX 25 KB)

Supplementary file5 (DOCX 23 KB)

Supplementary file6 (DOCX 6545 KB)

## Data Availability

Summary data is presented in supplemental files and any original data available on reasonable request.
