# Supplementary material for: Microangiopathy in temporal lobe epilepsy with diffusion MRI alterations and cognitive decline
Source: Acta Neuropathol. 2024 Oct 8;148(1):49. doi: 10.1007/s00401-024-02809-8 (PMC11461556; doi:10.1007/s00401-024-02809-8)
Supplement: Supplementary file 1 — Supplementary file1 (DOCX 77 KB) [file 401_2024_2809_MOESM1_ESM.docx]

**Supplemental methods:**

**Method for sclerotic index**

Sections were scanned and uploaded onto NDP.view3 Web server (Hamamatsu Photonics). We used the sclerotic index (SI), a standard method used in vascular dementia to quantify degenerative small vessel pathology on H&E sections (Lammie, Brannan et al. 1997, Blevins, Vinters et al. 2021). The 20 most sclerosed or thickened arterioles in the white matter were measured for both their inner diameter and external diameter in two perpendicular axis representing the maximal and minimal dimensions as well as the diameter of the perivascular space (PVS) (Supplemental Figure 1A). The internal border was taken as the inner endothelial cell surface and the external, the basement membrane or adventitial border. The SI (1-internal/external diameter) was calculated for each vessel and the mean SI for each case recorded. In addition, the mean PVS diameter and mean ratio of perivascular space/external vascular diameter (PVS/EXT) was calculated. A pilot study of eight cases, recounted after an interval, showed good reproducibility of SI measures (interclass correlation coefficient of 0.869).

**Automated Immunohistochemistry**

The staining was performed by automated IHC/ISH stainers (Leica BondMax 3 and Ventana Discovery Ultra). Prior to the staining, optimization of the antibody dilutions were carefully carried out. Sections were loaded on the slide trays and immunohistochemistry performed by the specified auto-stainer with its pre-defined protocols, including dewax, rehydration, incubation with hydrogen peroxide, heat or enzyme induced epitope retrieval (please see table for details), application of the primary antibodies (please see table for details of dilutions), a post antibody treatment that prepares tissue for the penetration of the subsequent for the polymer reagent, incubation with polymeric horseradish peroxidase (HRP) conjugated secondary antibody, visualization of the complex with 3,3'-Diaminobenzidine (DAB), and haematoxylin counterstaining that detects cell nuclei. Stained sections underwent a dehydration and clearing process in an automated multi-stainer (Leica ST5020) followed by the cover-slipping in the automated cover-slipper (Leica CV5030).

**Double labelling immunofluoresence for SMA and PDGFRβ**

Using Ventana discovery ultra, deparaffinisation was carried out and incubation at 65°C for 4 minutes and Ventana EZ Prep applied to clean the slides. Then sections underwent pre-treatment cell conditioning using CC1 reservoir which was applied 3 times and the slides were left to incubate following each application and then inhibitor CM to block endogenous activity and prevent non-specific background staining applied. Antibody against α-SMA (clone DAKO1A4, dilution factor 1:500, 100μL per slide) and following this, antibody amplification and localisation occurred with secondary antibody Rabbit Antibody AMP and multimer HRP for 20 minutes incubation and Red 610 fluorophore was then applied for 16 minutes followed by a dual sequence inhibitor containing HRP conjugates to inactivate endogenous peroxidase and a neutralisation step to bind to these HRP conjugates. Manual application of antibody against PDGFRβ (clone PDGFRβ 3257a, dilution factor 1:50, 100μL per slide), amplification and secondary antibody Mouse Antibody AMP and multimer HRP was then incubated for 20 minutes, after which a fluorescent dye, FITC fluorophore was applied, and the slides were incubated for a further 20 minutes. Following this, 4',6-diamidino-2-phenylindole (DAPI) was applied for counterstaining.

**QuPath – Olig2+ and NeuN+ cell density**

Olig2+ and NeuN+ whole slide images (WSI) were analysed using QuPath v0.4.1 (Bankhead, Loughrey et al. 2017). First, haematoxylin and DAB colour deconvolution stain vectors were determined from WSI with the highest and lowest intensities for the respective stains, and subsequently applied. DAB-positive objects were segmented using the QuPath Cellpose extension. (<https://github.com/BIOP/qupath-extension-cellpose>) (Stringer, Wang et al. 2021, Pachitariu and Stringer 2022). The resulting detection objects were classified into Olig2+ or NeuN+ cells for their respective stains using an object classifier. Cell density was calculated by: Cell density (cells/mm^2^) = Number of Olig2+ or NeuN+ cells / Area of ROI.

**QuPath – COL4+ vessel density and diameter analysis (also supplemental Figure 1)**

Haematoxylin and DAB colour deconvolution was determined and applied in QuPath as above. To segment COL4+ vessels, a pixel classifier was trained and applied on the DAB deconvoluted channel to create objects. These objects were then dilated by 3 μm to merge any discontinuous vessel staining, and then eroded to their original size. From the resulting vessel objects, those smaller than 25 μm^2^ were discarded and holes filled. Vessel density was calculated by: Vessel density (vessels/mm^2^) = Number of COL4+ vessels / Area of ROI.

Vessel objects from each WM region were exported as GeoJSON objects from QuPath and processed using a Python script to measure the median diameter. This involves interpolating approximately 200 points on the object perimeter, calculating a line perpendicular to the tangent (normal line) at each of these points, validating if the lines to be within a 15° angle tolerance at both ends of the normal line, and measuring the length of valid normal lines. Only vessel objects with >20 normal lines had the median length of normal lines (vessel width/diameter) calculated (Supplemental Figure 1). As an example, in random cases the mean number of line intersect per vessel in the superior temporal gyrus WM core ROI were 68/COL4+ vessel in a TLE case and 76/COL4+ vessel in post-mortem control with mean number of vessels analysed of 538 (103-3640) in TLE and 3879 (1574-7904) in the control.

**Qupath – SMA semi-automated method for vessel density and diameter analysis (see also Supplemental Figure 4)**

SMA stained slides were imported into QuPath (Bankhead, Loughrey et al. 2017), intensity thresholds set enabling optimal detection of vascular structures as for COL4. As SMA labelling was discontinuous in many vessels walls automated detection was observed in minority of vessels overcounted these as multiple, separate smaller vessels. Therefore, an additional manual step was added to the automated threshold, overriding the counts and re-classifying vessels as type 1 (with circumferential SMA) or type 2 (discontinuous SMA) with measurements of mid-tangential diameter of each vessel to assess the calibre. Due to this more time-intensive manual step in a pilot study of 5 cases, analysis of the six large ROI (superior temporal gyrus core and deep WM, middle temporal gyrus core and deep WM, inferior temporal gyrus core and deep WM, equivalent to the COL4 ROI analysis) was compared to an analysis of smaller ROI in each gyrus through random placement of rectangles of 0.2mm^2^ (equivalent to 10-20% of the total ROI area). Bland Altman plots (Graph pad prism 9) showed good limits of agreement between measures of vascular density (Bias 0.19, 95% limits of agreement (-5.6 to 6.05), and significant correlation (r=0.7, p=0.0003. Spearman’s rank correlation) between the two regions of interest. Therefore small ROI analysis for SMA was conducted across the whole series.

**PDGFRβ analysis, vascular structures and glia:**

PDGFRβ highlighted pericytes and perivascular cells in addition to small multipolar parenchymal cells not associated with vessels. PDGFRβ was therefore manually quantified for vascular structures in addition to automated labelling index. PDGFRβ-stained digital images were uploaded to NDP.view2 (Hamamatsu Photonics K.K.) and PDGFRβ+ vascular structures of all size with a visible lumen were classified into Type 1 or Type 2 vessels, according to either complete or partial vascular PDGFRβ+ pericyte coverage respectively (Figure 2K,M, Supplemental Figure 1B).

Rectangular ROI of 1-2mm^2^ were drawn at low power within the core and deep white matter of the STG and MTG in cases and controls and the density and mean vessel diameters calculated. In addition, vessels were further classified based on calibre as small capillary (<5 microns), capillaries (5 to ≤10 microns), small arterioles (10 to ≤25 microns) and arterioles (>25 microns) and the density and proportion of vessel types in each ROI calculated. This sampling method was developed following a pilot study for statistical reproducibility.

**Manual method for evaluation of PDGFRβ positive vessels**

Counting rules were developed to include all vessels with peripheral PDGFRBeta labelling whether in cross section or longitudinal section. A vessel needs to have a lumen by definition but may not have a pericyte or endothelial cell nucleus, particularly if in cross section. For vessels with branch points, it was counted and measured at one point but vessels not in continuity counted as separate vessels. Vessels crossing the margins of the counting box were included. Diameters were measured in the middle but at the narrowest point if in tangential section. Type 1 vessels were defined as those with a complete circumference of PDGFR labelling and Type 2 vessel had gaps or intermittent coverage. Single parenchyma cells without a lumen were not counted. In a pilot study of four TLE cases the entire white matter region was compared to analysis of varying random placement of counting boxes within large ROI using the NDP.view2 software. Good repeatability was achieved with 1.5mm^2^/per ROI on recounts; repeatability coefficient type 1 vessel density and diameter (0.92, 0.82) and type 2 vessel density and diameter (0.86, 0,88).

**Gene expression data with nanostring**

Samples from the temporal white matter were obtained from the 36 cases and total RNA isolated. For this study the deep and core white matter was not separately analysed. In brief, for each case, total RNAs were isolated from 1g of frozen tissue using RNAeasy kit (Qiagen, Hilden, Germany) according to manufactures’ instructions. The quality and concentration of total RNA was assessed using NanoDrop ND-1000 spectrophotometer (NanoDrop Technologies, Willmington, DE) and Agilient 2100 Bioanalyzer (Agilent Technology, Palo Alto, CA). Samples with a concentration above 55ng/ul, purity at 260/280nm is 2.0 or above, integrity RNA > 5 were used in the study. RNA lysates of each case were processed for Nanostring nCounter analysis using NanoString Neuropathology Gene Expression panel (XT Hs NeuroPath CSO; NanoString Technologies, Washington, USA), which target genes involved in neuroplasticity, development and aging, neuroinflammation, metabolism and maintenance of structural integrity pathways, and included eight housekeeping genes, six positive and eight negative control probes.

Nanostring nSolver software version 4.0 (NanoString, Washington, USA) was used to analyse gene expression data. Data were normalised using the geometric mean of housekeeping probes from NanoString (AARS, ASB7, CCDC127, CNOT10, CSNK2A2, FAM104A, LARS, MTO1, SUPT7L, TAD2B) and values were log transformed. Visualisation of data through constructions of heatmaps, correlative matrices and graphs were achieved using nSolver. Multivariate linear models using lm functions were performed to identify significant differentially expressed genes in different comparative groups based on Pathology (HS and NL), Age of Surgery (Under or Over 40yrs), Tissue Type (Surgical or PM). Benjamini-Hochberg adjusted P values were presented where available, otherwise unadjusted P values were noted. P< 0.05 is considered statistically significant. Pathway analyses were performed based on Gene Set analysis score, which is based on the extent to which a pathway is up or down regulated relative to the comparison group, taking to account t-statistics. Plots were generated from Pathways scores to show variations between HS and NL surgical (and PM) groups or between under and over 40 years at age of surgery.

**Diffusion MRI analysis**

The study included 24 patients with pre-operative DWI, including 10 cases with gene expression data) (Table 1). DWI was compared to 70 healthy controls acquired from a subset of a previous study (Vos, Winston et al. 2020). Diffusion data were collected as described below.  Mutli-shell DWI (2 mm isotropic resolution, gradient directions: 11, 8, 32, and 64 at b-values: 0, 300, 700, and 2500 s/mm2; ∂/Δ=21.5/35.9 ms, TE/TR=74.1/7600 ms) were collected on a 3T GE Discovery MR750. Images were denoised (Veraart, Novikov et al. 2016), Gibbs-unringed (Kellner, Dhital et al. 2016), corrected for signal drift (Vos, Tax et al. 2017), and distortion corrected using a synthesized b0 for diffusion distortion correction (Synb0-DisCo) produced from a T1-weighted MRI (Schilling, Blaber et al. 2019) which was fed into FSL’s top up (Andersson, Skare et al. 2003). Eddy currents and movement artifacts were corrected using FSL’s eddy tool (Andersson and Sotiropoulos 2016), rotating the b-vectors (Leemans and Jones 2009). Additionally, bias-field correction was performed in MRtrix and DWI images were interpolated to 1mm isotropic resolution.

**Pathology alignment**:

Structural 3D T1-weighted (MPRAGE; 1mm isotropic resolution) MRI images were registered to DWI using rigid transformation in NiftyReg. DWI were rotated to align the MTG flat and T1-weighted images aligned with histology sections, using macroscopic images and NeuN stained sections by three observers independently (LB, MT, SP, two post-doctoral scientists and one experienced consultant neuropathologist); if there was any disagreement between the MRI slice and histology the case was discussed and agreement reached on the best match. Using Freesurfer (version 7, (Fischl 2012)) the WM was parcellated and split into superficial and deep STG, MTG and ITG regions. The image was then rotated back to the diffusion image to extract the median tensors, including the mean diffusivity (MD) and fractional anisotropy (FA) for average water molecules mobility and diffusion anisotropy, respectively in addition to axial diffusivity (AD), radial diffusivity (RD) was obtained from the matched slice in addition adjacent three on either side (averaged over 7 slices) and Z scores calculated relative to control values.

**Fixel-based analysis (FBA):**

After preprocessing, fibre orientation distribution functions (ODFs) were computed using multishell multi-tissue constrained spherical deconvolution (MSMT-CSD) (Tournier, Calamante et al. 2004, Jeurissen, Tournier et al. 2014) with a group-averaged response function for white matter, grey matter, and cortical spinal fluid. A healthy population template was created by using white matter ODF images from 20 randomly selected ‘healthy’ control participants, using an interactive registration and averaging approach (Raffelt, Tournier et al. 2011). Spatial correspondence was achieved by registering white matter ODF images from all subjects to this template using ODF-guided non-linear registration (Raffelt, Tournier et al. 2011, Raffelt, Tournier et al. 2012). Using probabilistic tractography on the population template image, a whole-brain tractogram was generated comprising 20 million streamlines, which were then filtered to 2 million streamlines using the SIFT algorithm to reduce reconstruction biases (Smith, Tournier et al. 2013). An FBA framework was applied (Raffelt, Tournier et al. 2017); ‘fixel’ refers to a specific fibre population within a single image voxel. Different voxels within an image may contain a different number of fixels. These are computed based on segmentation of the WM ODFs with correspondence established between subject-specific fixels and those of the WM ODF template (Raffelt, Tournier et al. 2017). We utilised fibre density and cross-section (FDC) metric which was obtained at each white matter fixel in the population template space for all subjects. This metric combines microstructural and morphological information, such that it is sensitive to changes in the density of fibres passing in a particular direction within a given voxel, as well as the changes in the cross-section of fibre bundles that traverse multiple image voxels. Connectivity-based smoothing was performed on fixel-based measures for all participants (Raffelt, Smith et al. 2015).

**Clinical data and neuropsychometry**

Patients underwent a comprehensive set of routine neuropsychometric tests pre-operatively. This battery has been described previously (Baxendale and Thompson 2020) and complete datasets were available for 43 patients. Since the assessments spanned over two decades, the test versions were not uniform across the sample. The National Adult Reading test (NART) was used to assess pre-morbid intelligence (Bright, Hale et al. 2018). Test of Premorbid Functioning (TOPF) was used in place of the NART for 9 patients (Davis, Finch et al. 2009). In cases where the patients were unable to complete the NART or the TOPF, pre-morbid reading level was assessed using the Schonell Graded Word Reading test (Schonell 1942). All the patients were administered the Weschler Adult Intelligence Scale (WAIS). Verbal Intelligence Quotient (VIQ) from WAIS-3 and WAIS-Revised (Weschler, 1981), and Verbal Comprehension Index (VCI) from WAIS-4 were selected as measures of verbal cognition (Wechsler 1955, Wechsler 1981, Wechsler and Psychological 1997). The Digit Span subtest was selected as a measure of working memory. The discrepancy between reading IQ (as the estimate of optimal level of function) and current level of function (as assessed by the WAIS verbal ability scores) was used to determine deterioration in the preoperative patients. Patients were binarized into “decline” or “stable” groups based on their pre-surgical verbal cognitive abilities. A discrepancy of 10 or more between the estimated pre-morbid intelligence and VIQ/VCI was considered clinically relevant cognitive decline (Ohi, Sumiyoshi et al. 2017). Pre-operative working memory performance was similarly binarized as “impaired” or “unimpaired” A scaled score of <7 on Digit span was considered as impaired.

**Statistical analysis**

Statistical analysis was conducted using SPSS (IBM corporation, Version 29) and Graph Pad Prism software for graph constructions. Non-parametric tests were used for comparison of pathology variables between TLE cases and control groups and Wilcoxon signed rank test was used for differences between superficial and deep white matter for pathology and DWI variables. Bland Altman plots were used in pilot studies for the analysis of SMA vasculature. For RNA analysis, Benjamini-Hochberg adjusted P values were presented as shown, otherwise unadjusted P values were noted ; p< 0.05 was considered statistically significant. Pathway analyses were performed based on Gene Set analysis score, taking to account t-statistics and plots were generated from pathways scores to also show variations between HS and non-HS groups and age of surgery (less or older than 40 years ; further detail in supplemental methods). A logistic regression analysis was used for comparison of pathology variables between TLE groups binarized as with and without neuropsychological impairments using imputation for missing variables ; a multiple regression analysis was applied for any significant variables (p<0.05) also factoring for age at surgery. For comparison of DWI and pathology comparisons (and Z scores compared to healthy control data) univariate linear regression analysis was conducted, with significance taken at p<0.05.

References

Andersson, J. L., S. Skare and J. Ashburner (2003). "How to correct susceptibility distortions in spin-echo echo-planar images: application to diffusion tensor imaging." Neuroimage **20**(2): 870-888.

Andersson, J. L. R. and S. N. Sotiropoulos (2016). "An integrated approach to correction for off-resonance effects and subject movement in diffusion MR imaging." Neuroimage **125**: 1063-1078.

Bankhead, P., M. B. Loughrey, J. A. Fernandez, Y. Dombrowski, D. G. McArt, P. D. Dunne, S. McQuaid, R. T. Gray, L. J. Murray, H. G. Coleman, J. A. James, M. Salto-Tellez and P. W. Hamilton (2017). "QuPath: Open source software for digital pathology image analysis." Sci Rep **7**(1): 16878.

Baxendale, S. and P. Thompson (2020). "The association of cognitive phenotypes with postoperative outcomes after epilepsy surgery in patients with temporal lobe epilepsy." Epilepsy Behav **112**: 107386.

Blevins, B. L., H. V. Vinters, S. Love, D. M. Wilcock, L. T. Grinberg, J. A. Schneider, R. N. Kalaria, Y. Katsumata, B. T. Gold, D. J. J. Wang, S. J. Ma, L. M. P. Shade, D. W. Fardo, A. M. S. Hartz, G. A. Jicha, K. B. Nelson, S. D. Magaki, F. A. Schmitt, M. A. Teylan, E. T. Ighodaro, P. Phe, E. L. Abner, M. D. Cykowski, L. J. Van Eldik and P. T. Nelson (2021). "Brain arteriolosclerosis." Acta Neuropathol **141**(1): 1-24.

Bright, P., E. Hale, V. J. Gooch, T. Myhill and I. van der Linde (2018). "The National Adult Reading Test: restandardisation against the Wechsler Adult Intelligence Scale-Fourth edition." Neuropsychol Rehabil **28**(6): 1019-1027.

Davis, A., W. H. Finch, M. Chang, D. E. McIntosh, B. A. Rothlisberg and S. Paulson (2009). "Demographic Variables, Premorbid Functioning, and CHC Ability on the Woodcock-Johnson Tests of Cognitive Abilities for Preschoolers." Archives of Clinical Neuropsychology **24**(5): 539-539.

Fischl, B. (2012). "FreeSurfer." Neuroimage **62**(2): 774-781.

Jeurissen, B., J. D. Tournier, T. Dhollander, A. Connelly and J. Sijbers (2014). "Multi-tissue constrained spherical deconvolution for improved analysis of multi-shell diffusion MRI data." Neuroimage **103**: 411-426.

Kellner, E., B. Dhital, V. G. Kiselev and M. Reisert (2016). "Gibbs-ringing artifact removal based on local subvoxel-shifts." Magn Reson Med **76**(5): 1574-1581.

Lammie, G. A., F. Brannan, J. Slattery and C. Warlow (1997). "Nonhypertensive cerebral small-vessel disease. An autopsy study." Stroke **28**(11): 2222-2229.

Leemans, A. and D. K. Jones (2009). "The B-matrix must be rotated when correcting for subject motion in DTI data." Magn Reson Med **61**(6): 1336-1349.

Ohi, K., C. Sumiyoshi, H. Fujino, Y. Yasuda, H. Yamamori, M. Fujimoto, T. Sumiyoshi and R. Hashimoto (2017). "A Brief Assessment of Intelligence Decline in Schizophrenia As Represented by the Difference between Current and Premorbid Intellectual Quotient." Front Psychiatry **8**: 293.

Pachitariu, M. and C. Stringer (2022). "Cellpose 2.0: how to train your own model." Nat Methods **19**(12): 1634-1641.

Raffelt, D., J. D. Tournier, S. Crozier, A. Connelly and O. Salvado (2012). "Reorientation of fiber orientation distributions using apodized point spread functions." Magn Reson Med **67**(3): 844-855.

Raffelt, D., J. D. Tournier, J. Fripp, S. Crozier, A. Connelly and O. Salvado (2011). "Symmetric diffeomorphic registration of fibre orientation distributions." Neuroimage **56**(3): 1171-1180.

Raffelt, D. A., R. E. Smith, G. R. Ridgway, J. D. Tournier, D. N. Vaughan, S. Rose, R. Henderson and A. Connelly (2015). "Connectivity-based fixel enhancement: Whole-brain statistical analysis of diffusion MRI measures in the presence of crossing fibres." Neuroimage **117**: 40-55.

Raffelt, D. A., J. D. Tournier, R. E. Smith, D. N. Vaughan, G. Jackson, G. R. Ridgway and A. Connelly (2017). "Investigating white matter fibre density and morphology using fixel-based analysis." Neuroimage **144**(Pt A): 58-73.

Schilling, K. G., J. Blaber, Y. Huo, A. Newton, C. Hansen, V. Nath, A. T. Shafer, O. Williams, S. M. Resnick, B. Rogers, A. W. Anderson and B. A. Landman (2019). "Synthesized b0 for diffusion distortion correction (Synb0-DisCo)." Magn Reson Imaging **64**: 62-70.

Schonell, F. J. (1942). "Backwardness in the Basic Subjects." British Journal of Educational Psychology **12**: 172-175.

Smith, R. E., J. D. Tournier, F. Calamante and A. Connelly (2013). "SIFT: Spherical-deconvolution informed filtering of tractograms." Neuroimage **67**: 298-312.

Stringer, C., T. Wang, M. Michaelos and M. Pachitariu (2021). "Cellpose: a generalist algorithm for cellular segmentation." Nat Methods **18**(1): 100-106.

Tournier, J. D., F. Calamante, D. G. Gadian and A. Connelly (2004). "Direct estimation of the fiber orientation density function from diffusion-weighted MRI data using spherical deconvolution." Neuroimage **23**(3): 1176-1185.

Veraart, J., D. S. Novikov, D. Christiaens, B. Ades-Aron, J. Sijbers and E. Fieremans (2016). "Denoising of diffusion MRI using random matrix theory." Neuroimage **142**: 394-406.

Vos, S. B., C. M. Tax, P. R. Luijten, S. Ourselin, A. Leemans and M. Froeling (2017). "The importance of correcting for signal drift in diffusion MRI." Magn Reson Med **77**(1): 285-299.

Vos, S. B., G. P. Winston, O. Goodkin, H. G. Pemberton, F. Barkhof, F. Prados, M. Galovic, M. Koepp, S. Ourselin, M. J. Cardoso and J. S. Duncan (2020). "Hippocampal profiling: Localized magnetic resonance imaging volumetry and T2 relaxometry for hippocampal sclerosis." Epilepsia **61**(2): 297-309.

Wechsler, D. (1955). Manual for the Wechsler adult intelligence scale.

Wechsler, D. (1981). Wechsler adult intelligence scale : WAIS-R manual.

Wechsler, D. and C. Psychological (1997). WAIS-III : administration and scoring manual : Wechsler Adult Intelligence Scale.
