# Supplementary material for: Microangiopathy in temporal lobe epilepsy with diffusion MRI alterations and cognitive decline
Source: Acta Neuropathol. 2024 Oct 8;148(1):49. doi: 10.1007/s00401-024-02809-8 (PMC11461556; doi:10.1007/s00401-024-02809-8)
Supplement: Supplementary file 2 — Supplementary file2 (DOCX 63 KB) [file 401_2024_2809_MOESM2_ESM.docx]

**Supplemental Table 1. Cases with details of epilepsy pathology including hippocampal sclerosis ILAE subtype, anti-seizure medications and cause of death.**

ILAE = International League Against Epilepsy, SGS = Secondary generalised seizures, also now termed focal to bilateral tonic-clonic seizure, Status = episode of status epilepticus recorded at some time-point prior to surgery, SEEG = stereo EEG including depth electrode investigations prior to resective surgery at variable time points. EBTB – is the identifier for the Edinburgh brain tissue bank. Outcome is detailed at 2 and 5 years follow up using the ILAE scheme for seizure-free class ; only the 2 year time point was statistically compared to pathology factors. ASM = anti-seizure medications which list the total number of drugs trialled prior to resection and the commonest medications taken for this cohort. Comprehensive historical ASM information was not available for all the post-mortem cases and therefore has not been included in this table or statistically analysed. Small vessel disease risk factors are added for the surgical cases in the pathology studies (Hypertension HTN, Diabetes mellitus DM) and where available for the post-mortem cases and any additional potential risk factors for vascular degenerative disease identified at autopsy, e.g. ischaemic heart disease (IHD) reported as the cause of death; history of smoking was not included as reliable information not available on all cases.
