# Supplementary material for: Microangiopathy in temporal lobe epilepsy with diffusion MRI alterations and cognitive decline
Source: Acta Neuropathol. 2024 Oct 8;148(1):49. doi: 10.1007/s00401-024-02809-8 (PMC11461556; doi:10.1007/s00401-024-02809-8)
Supplement: Supplementary file 3 — Supplementary file3 (DOCX 37 KB) [file 401_2024_2809_MOESM3_ESM.docx]

Supplementary Table 2: Pathology data and statistical analysis
