# Supplementary material for: Microangiopathy in temporal lobe epilepsy with diffusion MRI alterations and cognitive decline
Source: Acta Neuropathol. 2024 Oct 8;148(1):49. doi: 10.1007/s00401-024-02809-8 (PMC11461556; doi:10.1007/s00401-024-02809-8)
Supplement: Supplementary file 5 — Supplementary file5 (DOCX 23 KB) [file 401_2024_2809_MOESM5_ESM.docx]

Supplementary Table 4: Neuropsychometry data with pathology, logistic regression analysis
