# Supplementary material for: Microangiopathy in temporal lobe epilepsy with diffusion MRI alterations and cognitive decline
Source: Acta Neuropathol. 2024 Oct 8;148(1):49. doi: 10.1007/s00401-024-02809-8 (PMC11461556; doi:10.1007/s00401-024-02809-8)
Supplement: Supplementary file 6 — Supplementary file6 (DOCX 6545 KB) [file 401_2024_2809_MOESM6_ESM.docx]

**Supplemental Figures**


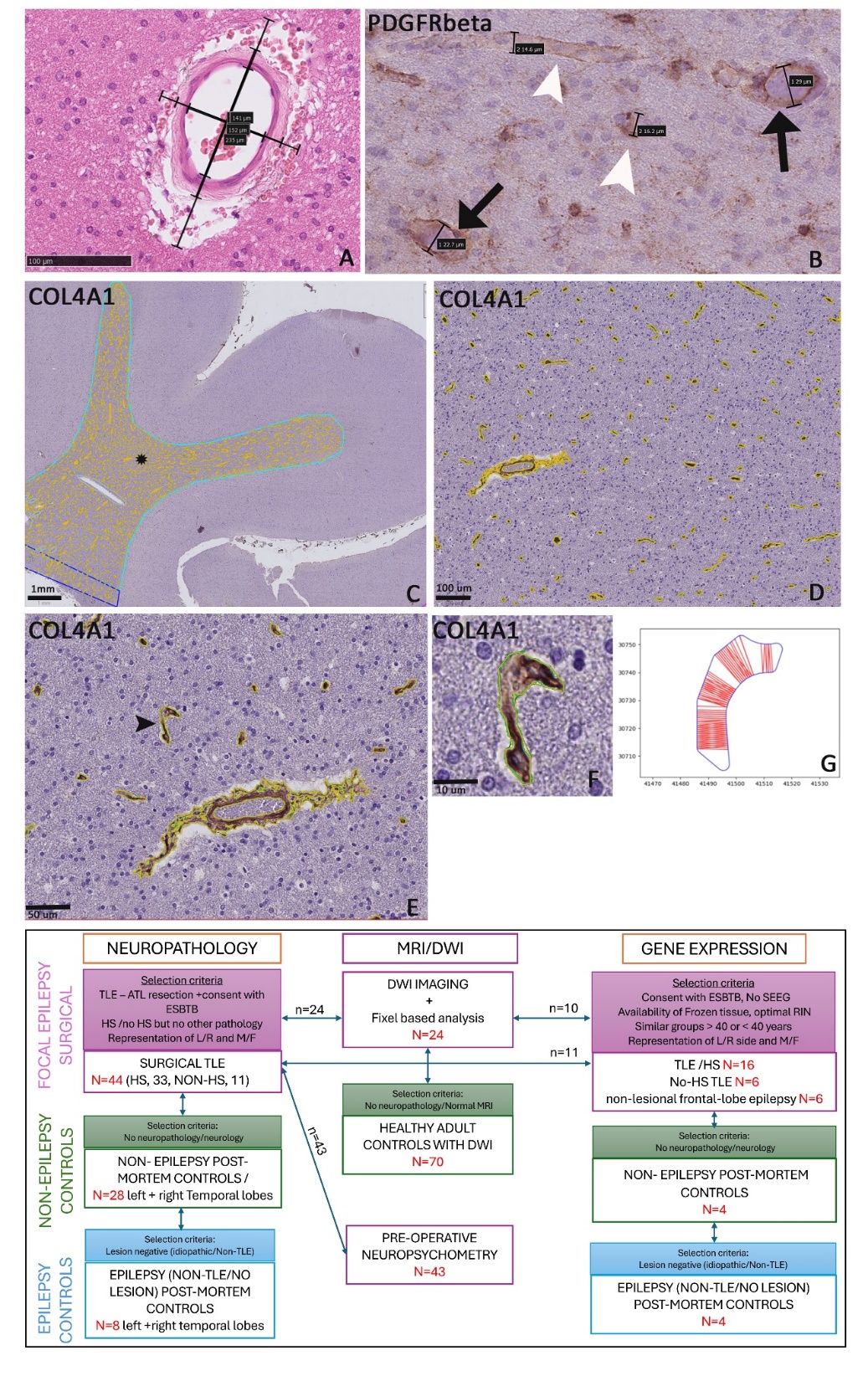


Supplemental Figure 1: Vascular measurements methods

(A). Sclerotic index measurement example of white matter vessel using NDP.view3 Web server (U13173-03, Hamamatsu Photonics K.K.). Twenty arterioles were selected for analysis, aiming to include most sclerosed vessels. Vessels in cross section were primarily selected and those in longitudinal section excluded. Many vessels were however in elliptical cross-section (as shown) and measures were therefore taken at both the shortest diameter and perpendicular to that axis and average values calculated. Internal diameters were measured using the endothelial borders, whereas external diameters taken from the basement membrane or adventitial borders and SI calculated (1 – (internal vascular diameter / external vascular diameter). The width of the perivascular space was also measured in the same axis as the vessel measurements.

(B). PDGRFβ labelling and measurements of diameters of small vessels (ranging from 14 to 29 microns diameter as shown) taken as the shortest midpoint axis. Vessels were also categorized as type 1 vessels (black arrows) with circumferential labelling or type 2 (white arrowheads) with discontinuous or intermittent labelling. Vessels were measured in longitudinal or cross-sectional axis and all non-contiguous, separate vascular structures were included. Single cells without capillary lumen were excluded.

(C). COL4 labeled vessels in the middle temporal gyrus core region. The asterisk in C is shown at higher magnification in Figures (D) and (E). COL4 detection using image analysis was used to outline and quantify the total number of vascular structures. All vessels with area greater than 25µ2, including irregularly shaped vessels (as shown with arrow in (E) and at higher magnification in (F)) had vessels diameter calculations using a python script generating random tangential intersects across the vessel lumen (as shown in a further example in (G)) from which the median value was calculated (note illustration in G does not correspond to the vessel shown in F).

Bottom of figure shows a diagram of cases selected in groups of TLE, epilepsy controls and non-epilepsy controls for the neuropathology (vascular and myelin analysis), MRI/ diffusion weighted imaging (DWI) and gene expression studies. The arrows indicate case numbers common t each study. Statistical comparisons were made between : (1) Neuropathology data and DWI data, (2) Gene expression and DWI data and (3) Neuropsychology data and pathology data. The core inclusion criteria for each group are also shown. The control cases used for the DWI, neuropathology and gene expression studies were different for each study. ATL = anterior temporal lobectomy, ESBTB = epilepsy brain and tissue bank, L/R = left/right, M/F= male/female, HS=hippocampal sclerosis.


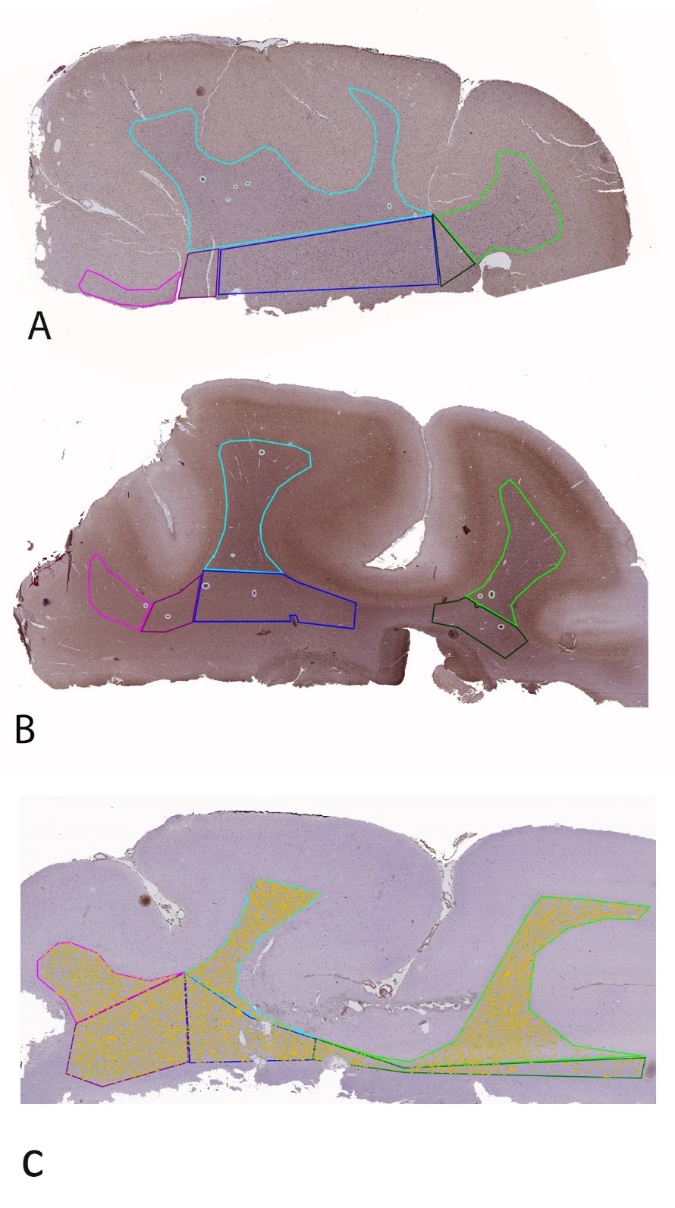


Supplemental Figure 2. White matter regions of interest

A-C. Examples of the white matter regions of interest (ROI) drawn with Qupath on sections labelled with PDGFRβ (A), neurofilament light chain (B) and COL4 (with vessel detection shown in yellow). Pink ROI is the superior temporal gyrus core ; deep white matter (purple ROI). Turquoise ROI is the middle temporal gyrus core ; deep white matter (blue ROI). Green ROI is the inferior temporal gyrus core ; deep white matter (dark green ROI). Tissue artefacts were excluded from analysis (grey lines).


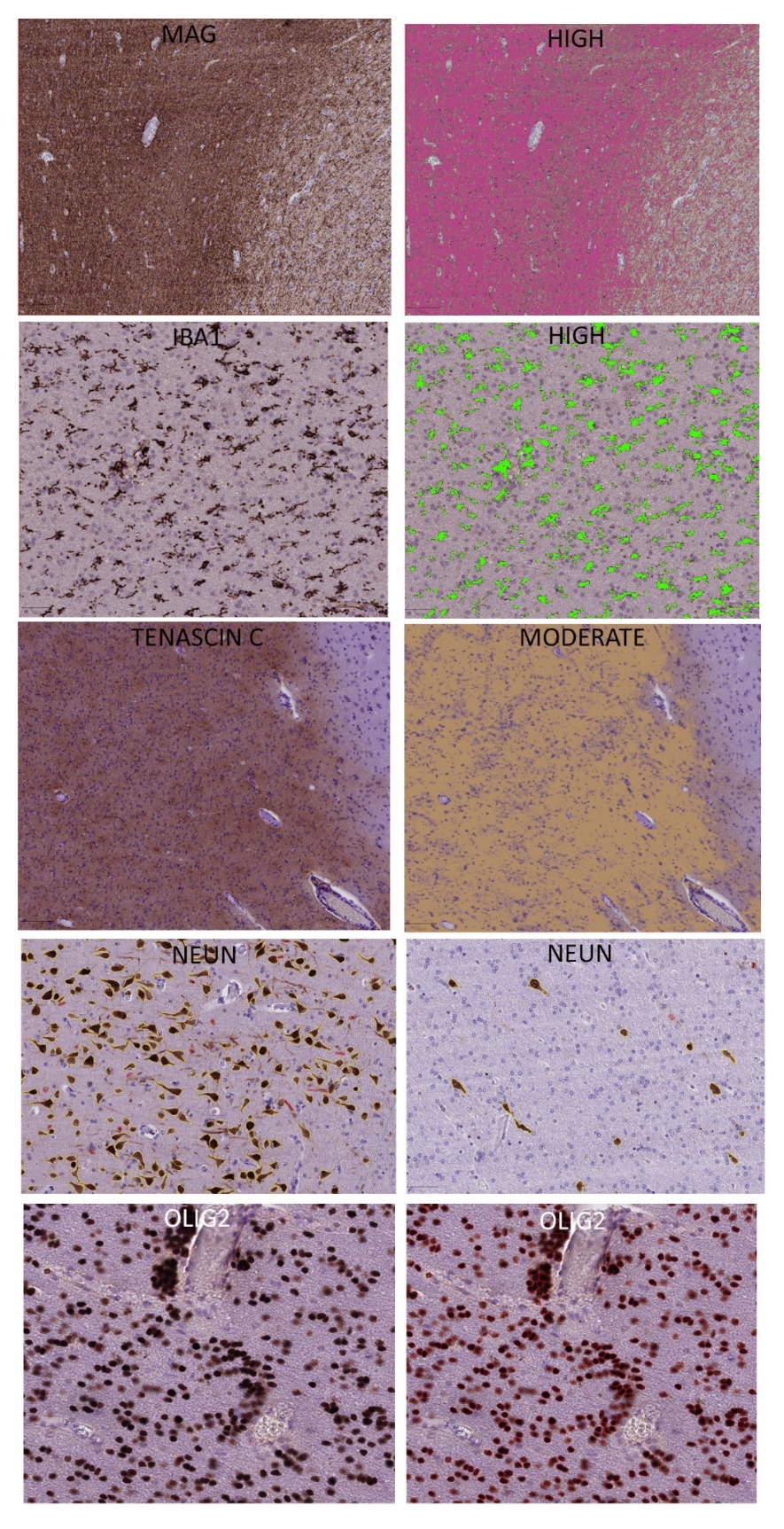


Supplemental Figure 3. Image analysis automated detection

Examples of automated detection of MAG, Iba1 and Tensacin C shown on the left with DAB staining and with different thresholds on the right side. Cell-pose method for estimation of neuronal number on NeuN stain is show in the cortex (left) compared to the white matter (right) with single, separate cells outlined in yellow. Cell-pose method for detection of white matter oligodendroglia is shown with OLIG2 on the bottom right (single cells outlined in red) with original DAB stain bottom left.


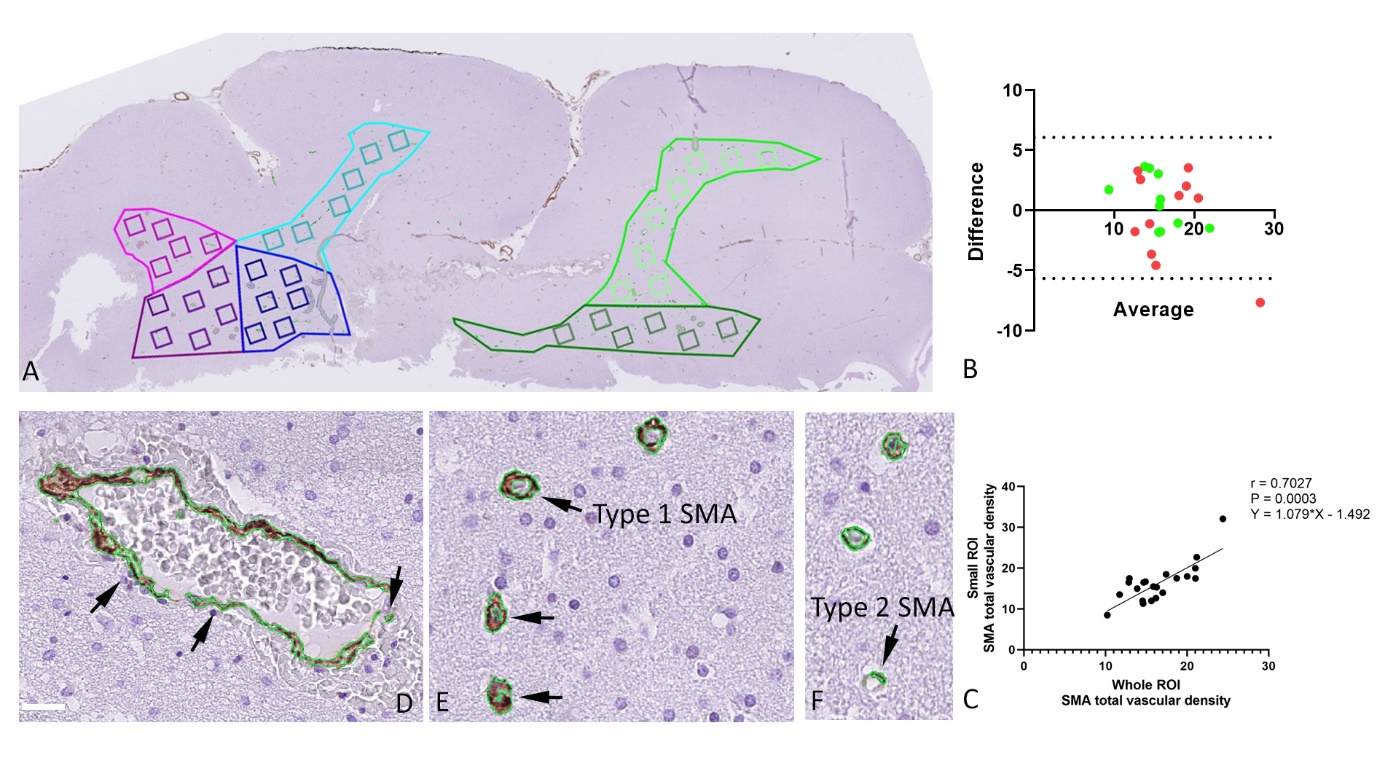


Supplemental Figure 4: Vascular measurements methods, SMA analysis

(A). For each large region of interest (ROI) of the core and deep white matter of the superior temporal gyrus (pink shades), mid temporal gyrus (blue shades) and inferior temporal gyrus (green shades) smaller regions of interest were randomly placed (each small box 0.25 mm2) to represent in total 10-20% of the large region area. In a pilot study of five cases (three TLE and 2 controls) comparison of vascular density data from evaluation of the larger ROI compared to small ROI showed good agreement using (B) Bland Altman plot (green dots = core, red dots = deep) and (C) Spearman’s correlation (see also supplemental methods). (D). SMA+ vessels with discontinuous labelling on automated detection using Qupath were occasionally overcounted as separate vascular structures and therefore an additional step tagging these vessels as (E) Type 1 (circumferential SMA) or (F) Type 2 (discontinuous SMA) was carried out and mean densities and vessel diameter calculated for each region.


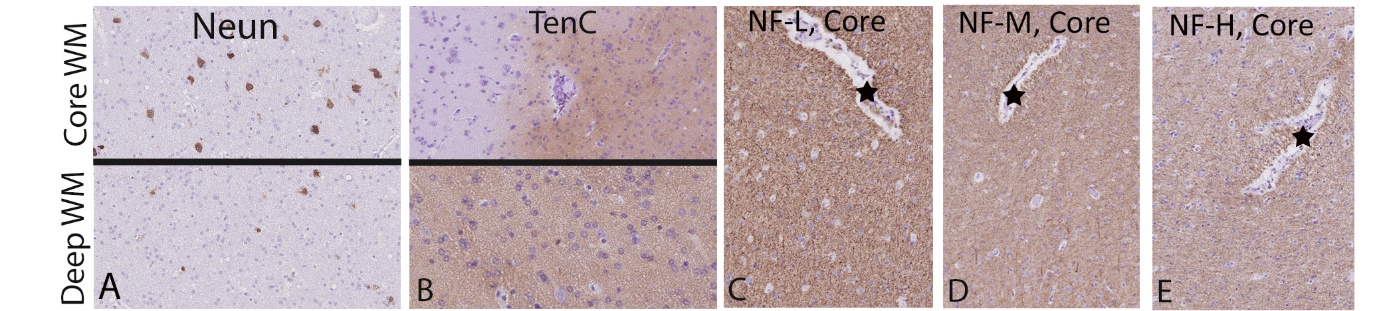


Supplemental Figure 5: NeuN, Tenascin and neurofilament labelling

(A). NeuN labels single interstitial neurons in both core and deep white matter in TLE and control groups. (B). Tenascin C shows predominant expression in the white matter (lower figure), focally extending into the cortex around blood vessels and glial cells (top figure). (C). Relatively uniform patterns of axonal labelling were noted in core and deep white matter in TLE with neurofilament markers (compared to myelin markers) for neurofilament light chain NF-L, (D) medium (NF-M) and (E) heavy chain (NF-H); these are shown on serial sections with the same vessel indicated with an asterisk in each figure.


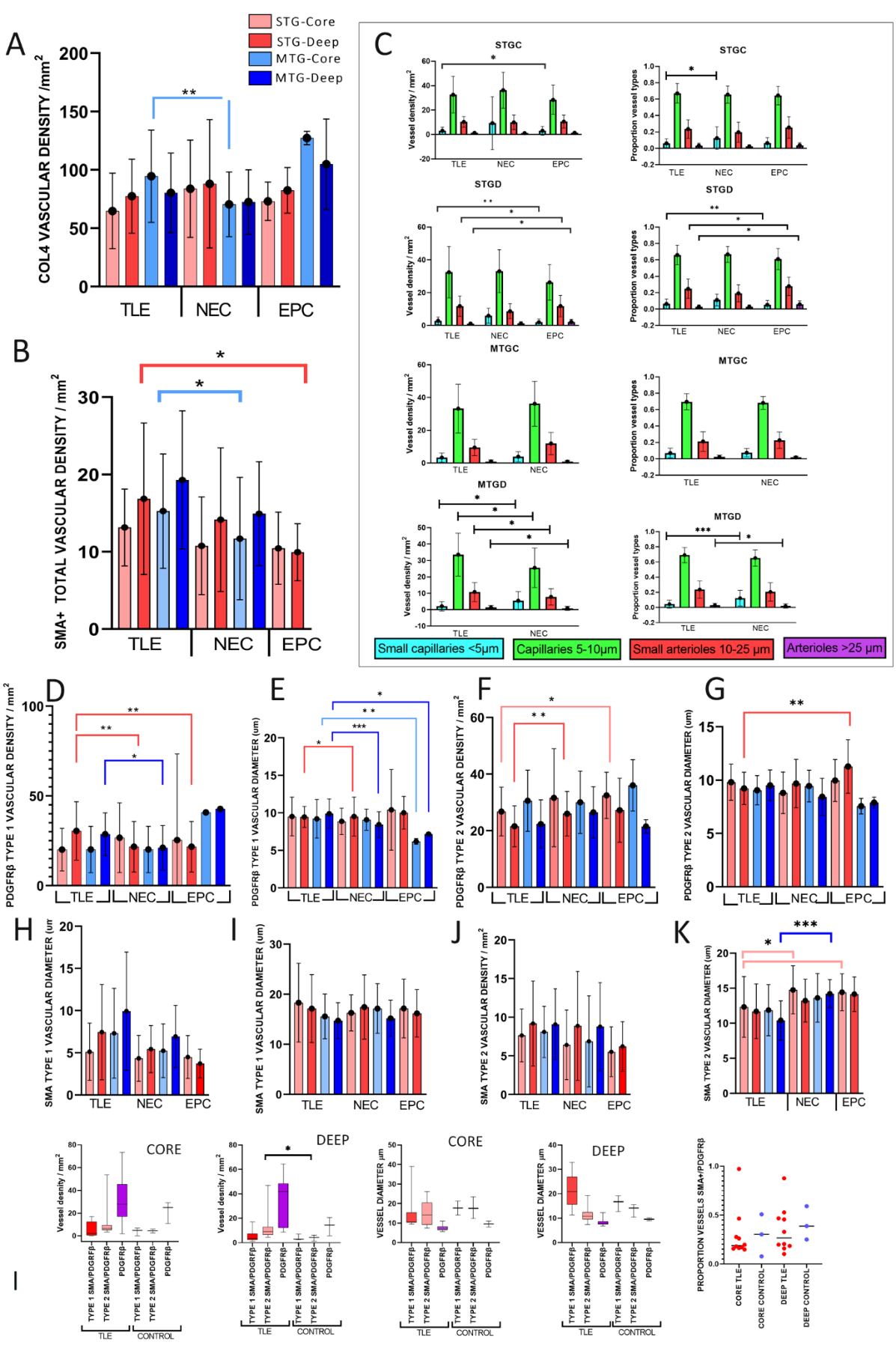


Supplemental Figure 6: Vascular measurements results

(A) COL4 vascular densities / mm2 and (B) SMA total vascular density /mm2 (standard deviation in error bars) for all regions of interest/ROI (Superior temporal gyrus (STG) core and deep white matter (red shades) and middle temporal gyrus (MTG) core and deep white matter (blue shades)) in TLE, non-epilepsy controls (NEC) and epilepsy control (EPC) groups. (C) PDGFRβ vessel types : Vessels were categorized into small capillaries (less than 5 microns diameter), capillaries (5-≤10 microns diameter), small arterioles (10-≤25 microns diameter) and arterioles (> 25 microns diameter) in superior temporal gyrus core and deep white matter (STGC, STGD) and middle temporal gyrus core and deep white matter (MTGC, MTGD) and the relative density (graphs on left) and proportion (graphs on right) of vessel types calculated in TLE, non-epilepsy controls (NEC) and epilepsy control groups (EPC). In the superior temporal gyrus: Kruskal Wallis non-parametric tests between groups showed higher ultra-small capillary densities and relative proportions in the NEC group in the STGC (p=0.03) and STGD white matter (p=0.007) with higher small and larger arteriole measures in the STGD in the EPC group (p=0.04). In the middle temporal gyrus, differences were only observed in the MTGD between groups with lower ultra-small capillary density and proportions (p= 0.01) but higher capillary (p=0.04), small arteriole (p=0.035) and larger arteriole densities (p=0.018) in the TLE than NEC group. The bars with two arms represent Mann-Whitney tests (two groups as shown) and one arm Kruskal Wallis (for three groups). (D-G). PDGFRβ vessel densities and diameters classified as type 1 or type 2 vessels in ROI between the three groups (see also data in Supplemental Table 2). (H-K). SMA vessel densities and diameters classified as type 1 or type 2 vessels in ROI between the three groups (see also data in Supplemental Table 2). (L). Double label SMA/PDGFRβ quantitative analysis: Vessels in ROI in the core or deep white matter of STG and MTG were counted in the eight cases as: SMA+/PDGFRβ+ (type 1) with continuous SMA peri-vascular coverage or SMA+/PDGFRβ+ (type 2) with incomplete SMA coverage. Vessels only PDGFRβ+ were counted but SMA+/ PDGFRβ- vessels were not detected. The diameter of each counted vessel was also measured. Data is shown graphically with significantly higher type 2 vessels in TLE cases. The proportion of double-labelled vessels PDGFRβ+/SMA+ was lower in TLE (p=0.028 Mann Whitney test). Significance shown in graphs * p<0.05, ** p <0.01 and *** p<0.001

Supplemental Figure 7. Pathology correlations


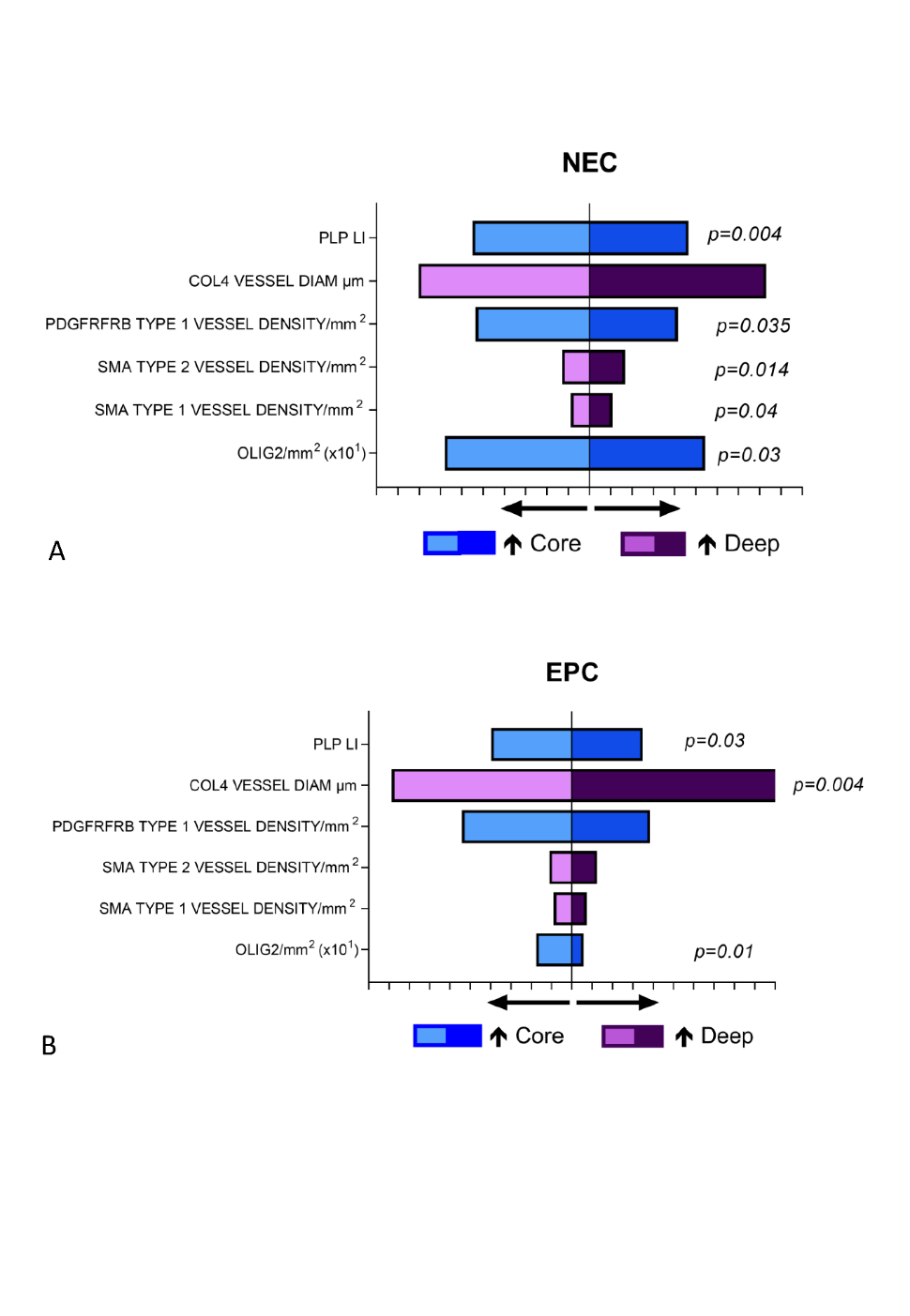


(A. B.) Pyramid graphical representation of pathology factors which showed significant differences between the superficial and deep white matter (data shown is average scores across all core and deep white matter regions) in non-epilepsy (NEC) and epilepsy control groups (EPC). Significance values shown as p values using Wilcoxon signed rank test.


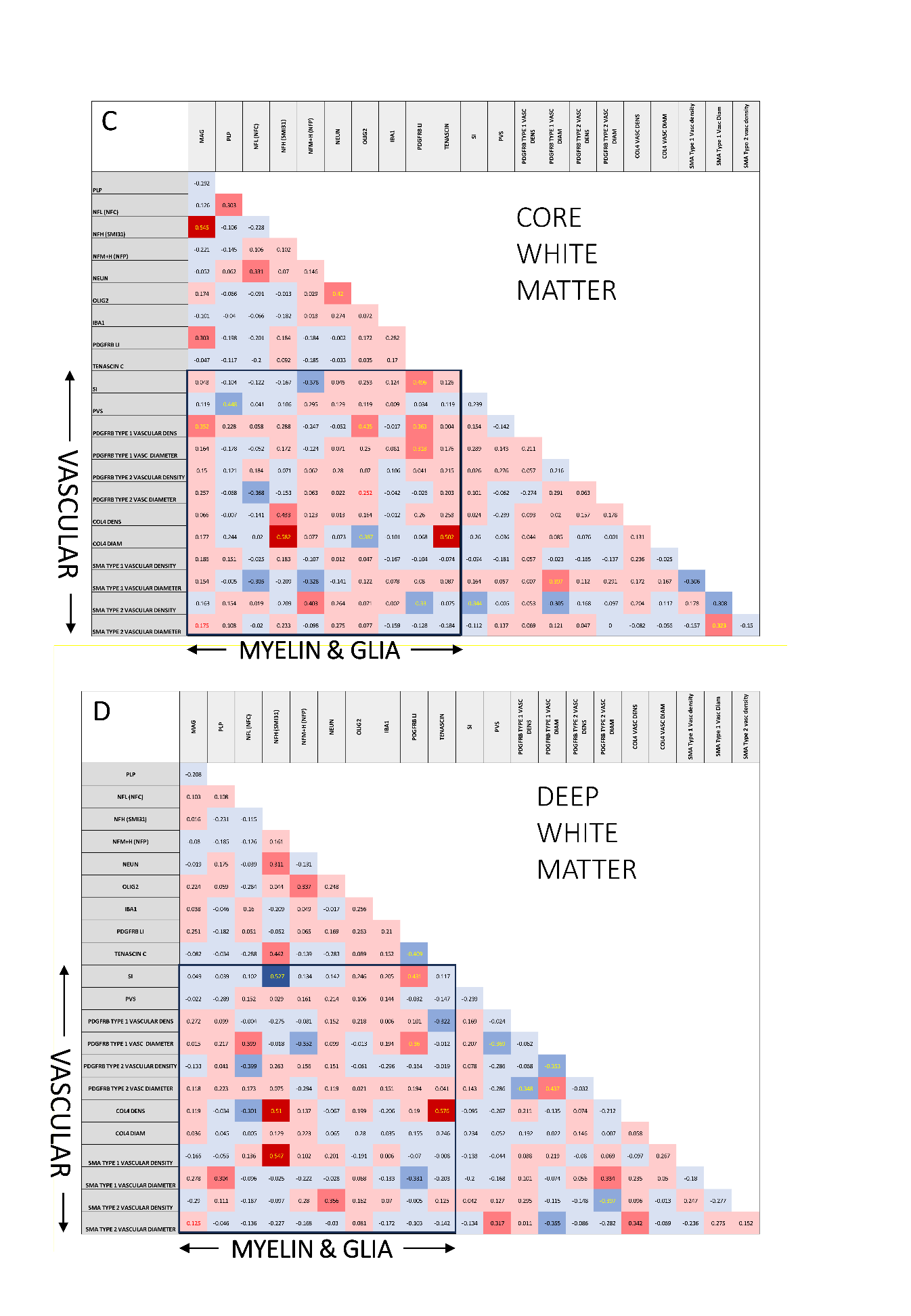


(C, D.) Heat Maps for Spearman’s correlations (r values shown in boxes) of pathology variable in Core (C) and Deep (D) white matter. Positive correlations shown in red and negative correlations in blue (darker colors for greater correlation coefficient). Significant values are shown in yellow font (p<0.05). There was an impression for clustering of vascular morphometry measures showing more frequent correlations with glial and myelin markers (outlined with black border) in both core and deep white matter.


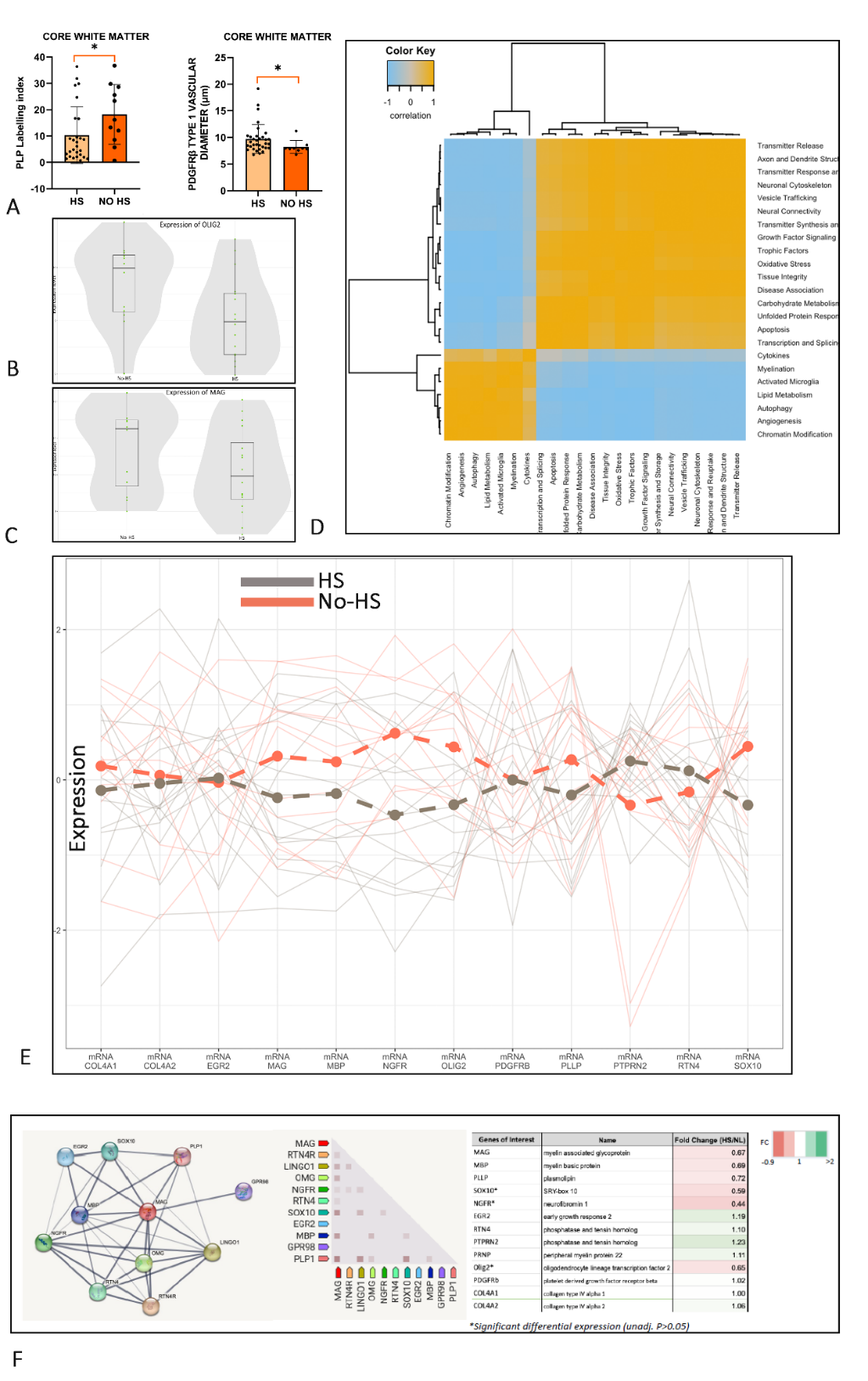


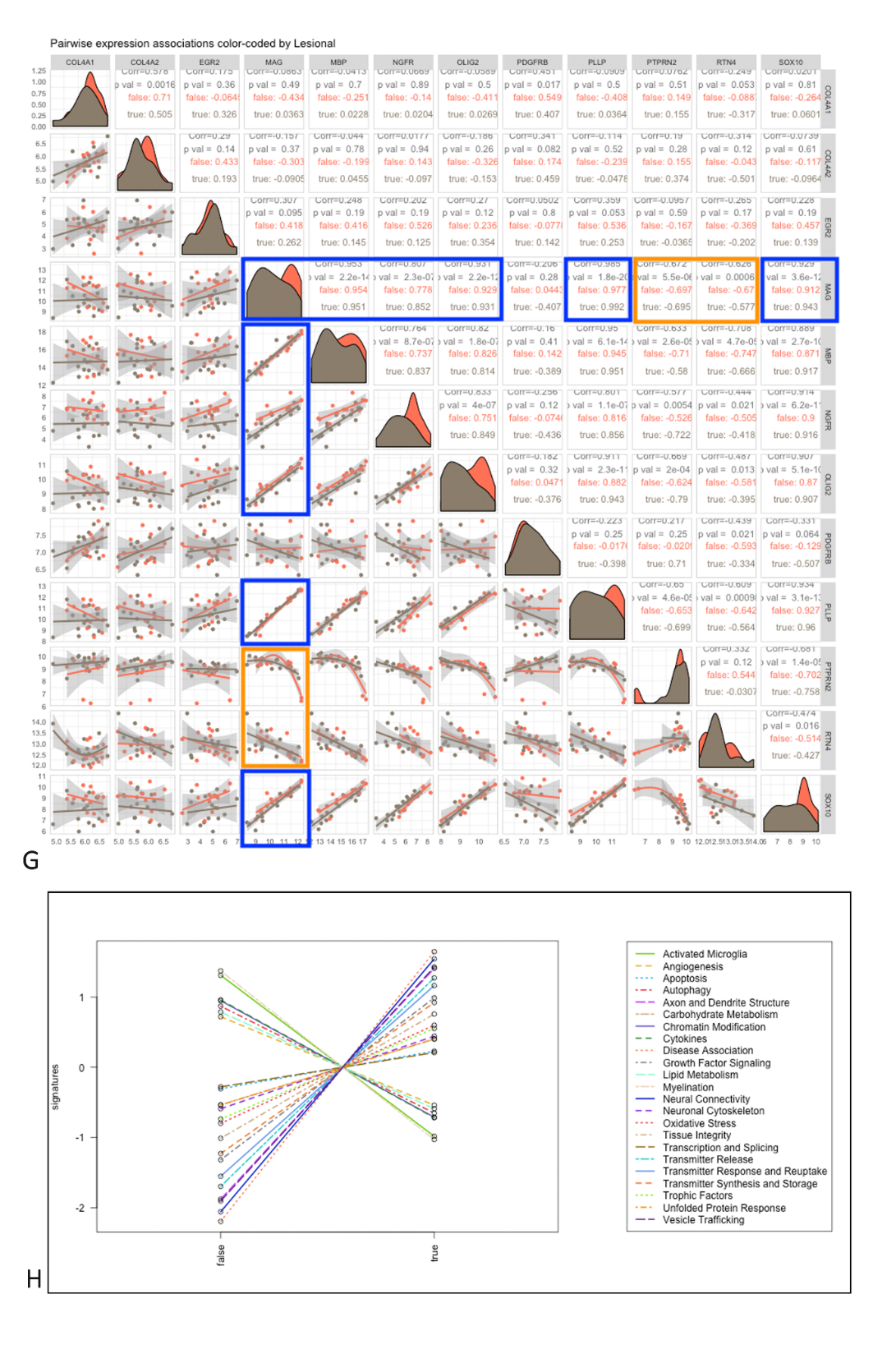


Supplemental Figure 8. Hippocampal sclerosis (HS) compared to no-HS cases

(A). Bar graphs of pathology measures in HS and non-HS cases for PLP and PDGFRβ Type 1 vascular diameter in the core white matter showing significant differences between groups (p<0.05); other pathology variables did not show a difference in deep or core white matter regions. (B) Gene expression showed reduced Olig2 and MAG (C) in deep white matter (p<0.05) in HS compared to non-lesional surgical epilepsy cases. (D). Heatmap of the correlation matrix of pathway scores indicating positive correlation (orange) between Angiogenesis and Myelination. (Blue = negative correlation).  (E and F). The expression of genes known to interact with MAG such as MBP, SOX10 and NGFR, were significantly reduced in HS cases compared to no-HS cases (P<0.05; refer to the line graph in (E) for a visualization illustration, and table in (F) for specific fold change reduction). The network map extracted from STRING DB (a functional protein association network database [66] (F, left) shows the close interaction between MAG and MBP, SOX10, NGFR. (G). Series of correlation plots to illustrate the distribution of MAG and gene of interests, and their relationships in HS (grey) and no-HS groups (red). A positive association was observed between MAG and genes associated with myelination and angiogenesis in HS and no-HS groups (P<0.001, blue boxes), including MBP (Pearson’s coefficient, HS, no-HS dataset; 0.951, 0.953), NGFR (0.853, 0.778), OLIG2 (0.931, 0.929), PLLP (0.992, 0.977), SOX10 (0.943, 0.12). PTPRN2 (-0.695, -0.697) and RTN4 (-0.577, -0.670) negatively correlated with MAG expression in HS and no-HS cases (P<0.001; orange boxes). (H). Covariate plots where pathways scores, calculated by nSolver, were plotted against pathology groups (HS ‘true’ or no-HS ‘false’). The graph indicated that the pathway scores for myelination, angiogenesis, activated microglia, autophagy, lipid metabolism, cytokines, chromatin modification were lower in HS cases compared to no-HS cases, while the opposite was observed by other pathways.


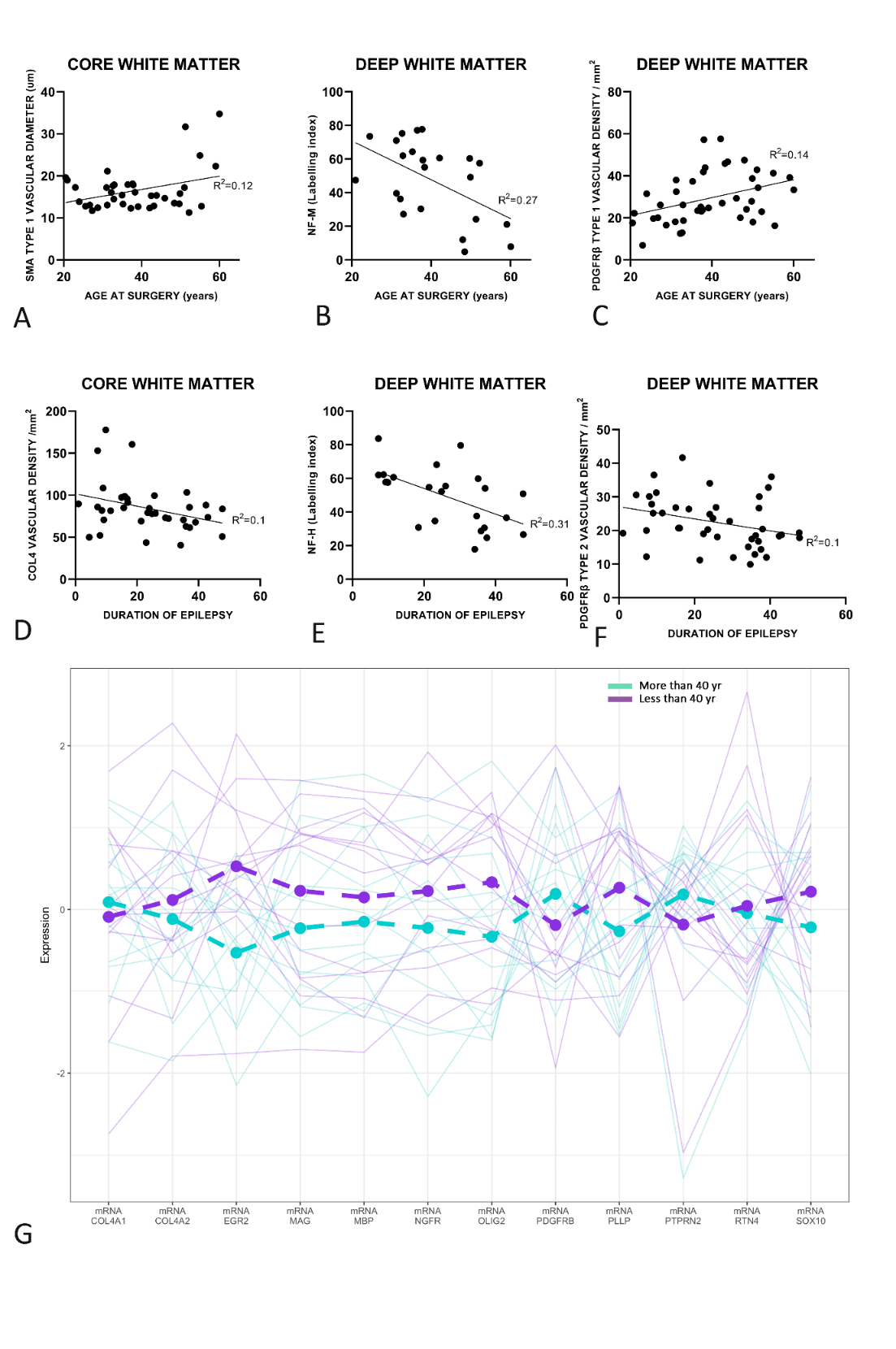


Supplemental Figure 9. Clinical correlations of pathology measures with age.

(A-C). Age at surgery: Correlated positively with SMA vascular diameter in the core white matter across all gyri (A) and with neurofilament-medium (NF-M) inversely in the deep white matter (B) whereas PDGFRβ type 1 vascular densities in the deep white matter positively correlated with age. (D-F). Duration of epilepsy: COL4 vascular density negatively correlated with duration in the core (D) and neurofilament-heavy chain labelling (NF-H) and (E) PDGFRβ type 2 vascular densities (F) in the deep white matter (all Spearman’s correlations, R2 value indicated on graphs, using graph pad statistical analysis ; p values <0.05 to 0.005). (G). Visual illustration showing the expression of targeted genes in cases with age of surgery over or under 40 years. The older cohort (green) show lower expression (p<0.05) of MAG, PLLP, OLIG2 mRNA compared to the younger cohort (purple) whereas the gene expression of PDGFRβ is higher in the younger cohort than the older cohort.
